# Defining the genetic susceptibility to cervical neoplasia—A genome-wide association study

**DOI:** 10.1371/journal.pgen.1006866

**Published:** 2017-08-14

**Authors:** Paul J. Leo, Margaret M. Madeleine, Sophia Wang, Stephen M. Schwartz, Felicity Newell, Ulrika Pettersson-Kymmer, Kari Hemminki, Goran Hallmans, Sven Tiews, Winfried Steinberg, Janet S. Rader, Felipe Castro, Mahboobeh Safaeian, Eduardo L. Franco, François Coutlée, Claes Ohlsson, Adrian Cortes, Mhairi Marshall, Pamela Mukhopadhyay, Katie Cremin, Lisa G. Johnson, Suzanne Garland, Sepehr N. Tabrizi, Nicolas Wentzensen, Freddy Sitas, Julian Little, Maggie Cruickshank, Ian H. Frazer, Allan Hildesheim, Matthew A. Brown

**Affiliations:** 1 Institute of Health and Biomedical Innovation, Queensland University of Technology, Translational Research Institute, Princess Alexandra Hospital, Woolloongabba, Australia; 2 Program in Epidemiology, Fred Hutchinson Cancer Research Center, Seattle, WA, United States of America; 3 Department of Population Sciences, Beckman Research Institute, City of Hope, Duarte, CA, United States of America; 4 Department of Pharmacology and Clinical Neuroscience, Umeå University, Umeå, Sweden; 5 Department of Public Health and Clinical Medicine, Umeå University, Umeå, Sweden; 6 Division of Molecular Genetic Epidemiology, German Cancer Research Center (DKFZ), Heidelberg, Germany; 7 Center for Primary Health Care Research, Lund University, Lund, Sweden; 8 Nutritional Research, Department of Public Health and Clinical Medicine, Umeå University, Umeå, Sweden; 9 MHC Laboratory for Cytopathology, Dr.Steinberg GmbH, Soest, Germany; 10 Department of Obstetrics and Gynecology, Medical College of Wisconsin, Milwaukee, Wisconsin, United States of America; 11 Division of Clinical Epidemiology and Aging Research, German Cancer Research Center (DKFZ), Heidelberg, Germany; 12 Division of Cancer Epidemiology and Genetics, National Cancer Institute, Bethesda, MD, United States of America; 13 Division of Cancer Epidemiology, McGill University, Montreal, QC, Canada; 14 Département de Microbiologie, Infectiologie et Immunologie, Université de Montréal, Montréal, QC, Canada; 15 Internal Medicine and Clinical Nutrition, Institute of Medicine, Sahlgrenska Academy University of Gothenburg, Gothenburg, Sweden; 16 Centre for Bone and Arthritis Research, Institute of Medicine, Sahlgrenska Academy, University of Gothenburg, Gothenburg, Sweden; 17 Regional World Health Organisation Human Papillomavirus Laboratory Network, Department of Microbiology and Infectious Diseases, The Royal Women’s Hospital, Parkville, Victoria, 3052, Australia; 18 Department of Obstetrics and Gynaecology, University of Melbourne, Murdoch Childrens Research Institute, The Royal Children’s Hospital, Parkville, Victoria, 3052, Australia; 19 Cancer Council NSW, Sydney, NSW, Australia; 20 Sydney School of Public Health, University of Sydney, Camperdown, NSW, Australia; 21 School of Public Health and Community Medicine, University of New South Wales, Kensington, NSW, Australia; 22 School of Epidemiology, Public Health and Preventive Medicine, Faculty of Medicine, University of Ottawa, Ottawa, Canada; 23 Division of Medical Education, University of Aberdeen, Aberdeen, Scotland; 24 Faculty of Medicine and Biomedical Sciences, University of Queensland, Translational Research Institute, Princess Alexandra Hospital, Woolloongabba, QLD, 4102, Australia; University College London, UNITED KINGDOM

## Abstract

A small percentage of women with cervical HPV infection progress to cervical neoplasia, and the risk factors determining progression are incompletely understood. We sought to define the genetic loci involved in cervical neoplasia and to assess its heritability using unbiased unrelated case/control statistical approaches. We demonstrated strong association of cervical neoplasia with risk and protective HLA haplotypes that are determined by the amino-acids carried at positions 13 and 71 in pocket 4 of HLA-DRB1 and position 156 in HLA-B. Furthermore, 36% (standard error 2.4%) of liability of HPV-associated cervical pre-cancer and cancer is determined by common genetic variants. Women in the highest 10% of genetic risk scores have approximately >7.1% risk, and those in the highest 5% have approximately >21.6% risk, of developing cervical neoplasia. Future studies should examine genetic risk prediction in assessing the risk of cervical neoplasia further, in combination with other screening methods.

## Author summary

Around 1% of women with cervical human papillomavirus (HPV) infection progress to cervical cancer. Previous studies had indicated that a person’s genetic makeup could predispose to HPV-associated cervical cancer, and that some of the genes likely to be involved include the immune-related human leukocyte antigen (HLA) genes among the major histocompatibility complex (MHC). However, it has been difficult to determine which alleles might be associated with cervical pre-cancer or cancer due to the complex and high level of co-inheritance of MHC alleles. Here, we performed a genome-wide association study that assessed the correlation of genetic variants among those with cervical cancer and healthy controls. We show that host genetics is a major determinant of HPV-associated cervical cancer, with 36% of liability due to common genetic variants in the population, and identify both risk and protective HLA alleles. Our study was also sufficiently powerful to identify particular residue variants on a number of the immune-related proteins that provide risk or protection, providing further insight into the biological basis for cervical cancer development. Our findings could lay the foundation for screening for people at increased risk of developing cancer following HPV infection, and aid in the treatment and prognosis of cervical cancer.

## Introduction

Cervical cancer remains a major cause of female mortality worldwide, particularly in developing countries that have limited screening programs [1]. Only a small fraction (~1%) of women with cervical human papillomavirus (HPV) infection go on to develop cervical neoplasia [2], and the factors determining risk of progression are incompletely understood. In the current study we used the hypothesis-free genome-wide association study (GWAS) approach to identify genetic variants associated with cervical neoplasia. These variants may underlie disease mechanisms and point to genetic markers of progression to cervical neoplasia.

A genetic contribution to the risk of HPV-associated cervical neoplasia is supported by several lines of evidence. A family segregation study suggested that shared genes account for 27% of cervical cancer heritability [3]. Also, persistent HPV infections are associated with the two genetic conditions: epidermodysplasia verruciformis caused by mutations in the *EVER1* and *EVER2* genes [4], and WHIM syndrome, associated with mutations in *CXCR4* [5]. Furthermore, genetic associations have been reported with HLA loci in cervical cancer in several studies using HLA typing and genome-wide association study (GWAS) approaches. Specifically the haplotype HLA-B*0702-DRB1*1501/HLADQB1*0602 is associated with increased disease risk, and reduced risk is associated with alleles of the haplotype HLA-B*1501/HLA-DRB1*1301/HLA-DQA1*0103/HLA-DQB1*0603 [6–10].

Resolving which alleles on these haplotypes are primarily associated with cervical pre-cancer and cancer is challenging due to the complex and extensive linkage disequilibrium that occurs across the major histocompatibility complex (MHC). Recently it has been suggested that the haplotypic associations with HLA-B*0702 and HLA-DRB1*1501/HLA-DQB1*0602 are largely driven by allelic variation in the *MICA* gene (rs67841474) and the effects of a SNP nearby *HLA-DRB1* that affects HLA-DRB1 expression (rs9272143) [9, 11]. Non-MHC associations with cervical cancer have also been reported with polymorphisms in a large number of genes from candidate gene studies, including *IRF3*, *TLR2*, *EXO1*, *CYBA*, *XRCC1* and *FANCA* [12], *OAS3*, *SULF1*, *IFNG*, *DUT*, *DMC1*, *GTF2H4* and *EVER1/2* [13], *ERAP1*, *LMP7* and *TAP2* [14], *TP53* [15], *TERT* [16] and *IL17* [17]. However, none of these findings have achieved genome-wide levels of significance, and as yet no non-MHC locus has been robustly associated with cervical pre-cancer or cancer. GWAS have been reported for cervical cancer in Scandinavian [9], Chinese [18] and Japanese cohorts [19]. Two non-MHC associations were detected in the Chinese study (rs13117307 at chromosome 4q12 within the gene *EXOC1*; rs8067378 at chromosome 17q12 upstream of the gene *GSDMB*). We report here a GWAS aiming to define genetic susceptibility to HPV-associated cervical neoplasia.

## Results

### Quality control

After quality control filters were applied, a total 2866 cases and 6481 controls remained. Details of these and the cohorts from which they originated are provided in Table 1. These were genotyped or imputed for 10,863,230 SNPs. Using logistic regression including 4 principal components as covariates, genome-wide association testing was performed. The genomic inflation factor (1000) was 1.02 (Q-Q plot S1 Fig).

**Table 1:** Characteristics of cervical neoplasia cases in participating study populations following quality control steps.

			Histology			Behaviour		HPV status			
Studies	Cases	Controls	Adeno-carcinoma	Squamous cell carcinoma	Other	In Situ	Invasive	HPV16 and -18	HPV16 no -18	HPV18 no -16	Negative HPV16 and -18
Montreal	95	0	0	NA	NA	NA	N/A	N/A	N/A	N/A	N/A
NCI [20]	194	0	97	88	N/A	57	137	N/A	38	14	93
NSW [21]	274	0	10	256	N/A	266	N/A	11	62	7	67
CerGe [22]	98	0	N/A	N/A	N/A	N/A	N/A	N/A	N/A	N/A	N/A
Seattle [7]	751	0	424	327	N/A	271	480	44	258	112	45
SUCCEED [20]	314	0	11	65	238	213	76	17	195	18	N/A
TOMBOLA[23]	324	0	N/A	N/A	N/A	N/A	N/A	16	115	15	177
Trimble[24]	94	0	N/A	N/A	N/A	N/A	N/A	N/A	N/A	N/A	N/A
Umea [25, 26]	722	1319	4	135	583	529	N/A	N/A	N/A	N/A	N/A
WTCCC [27]	0	5443	0	0	0	0	0	0	0	0	0
TOTALS	2866	6762	546	871	821	1336	693	88	668	166	382

N/A, not available.

### MHC findings

**Fig 1:**
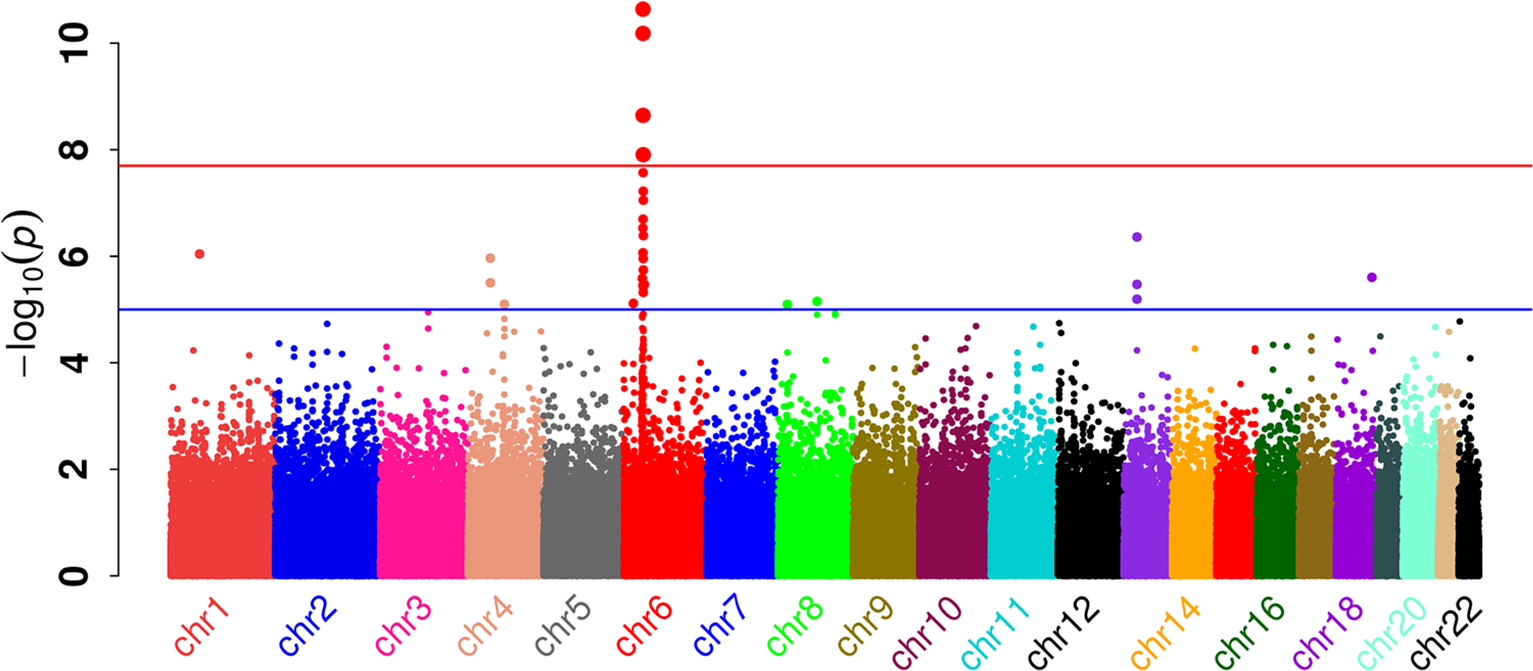
Manhattan plot of genome-wide association study of cervical neoplasia.

**Fig 2:**
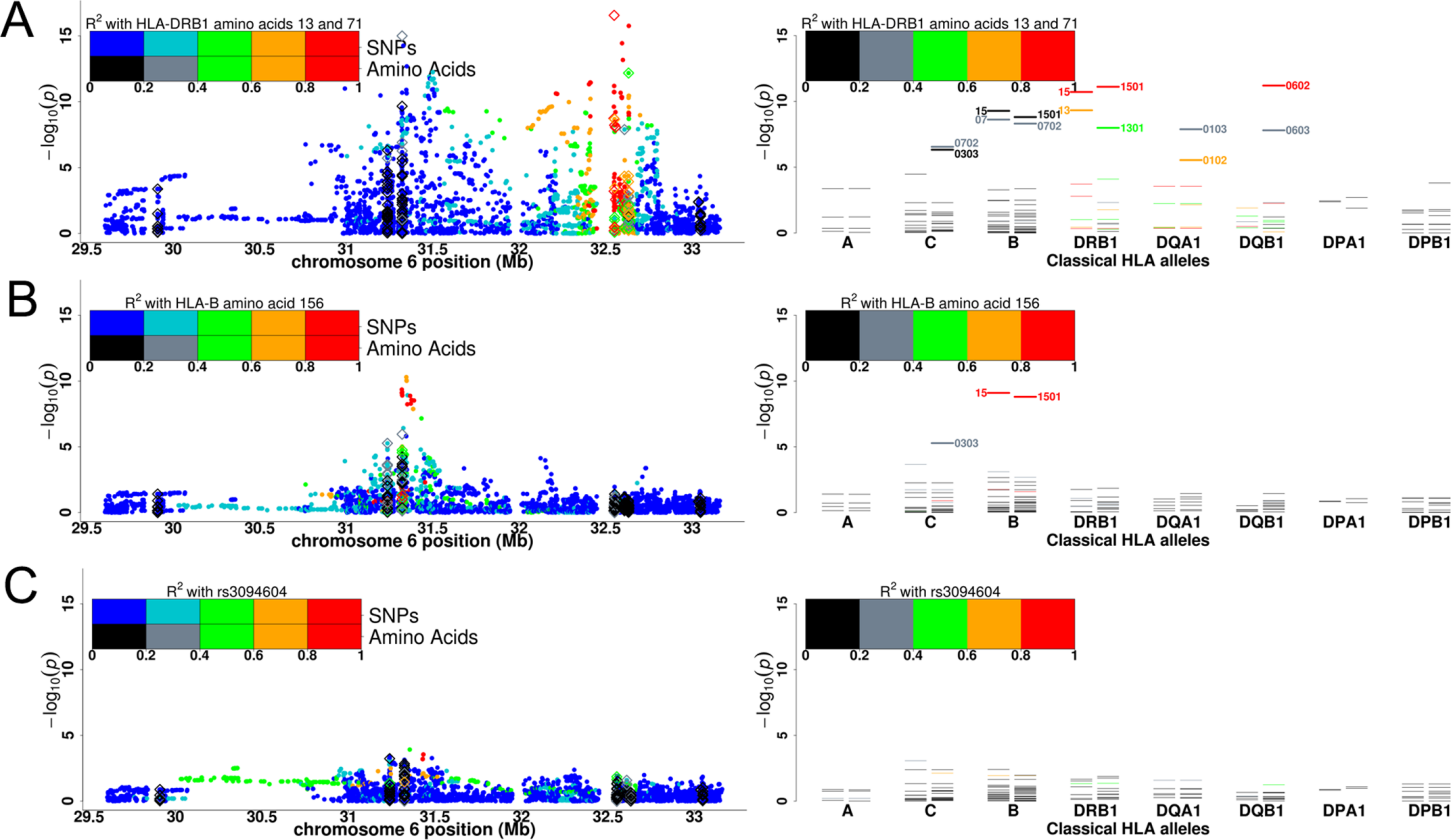
Zoom plot of the MHC showing association with cervical neoplasia. SNP associations are reported as filled-in dots, HLA amino-acid associations as hollow diamonds (*P*-values are for omnibus test of association at specific amino-acid positions). Colours represent extent of linkage disequilibrium with the HLA amino-acid(s) or SNP stated in the figure. (A) The linkage disequilibrium with amino acids at positions 13 and 71 that form part of the p4-pocket of HLA-DRB1. Reading from the p-telomere the HLA loci at which amino-acid constituents have been imputed are *HLA-A*, *-C*, *-B*, *-DRB1*, *-DQA1*, *-DQB1*, *-DPA1* and–*DPB1*. Allele-specific HLA type associations are given in the right hand plot. (B) Cervical neoplasia MHC association results having conditioned on amino acid positions 13 and 71 in HLA-DRB1. Linkage disequilibrium with the next largest association amino acid position 156 at HLA-B is shown. (C) Cervical neoplasia MHC association results having conditioned on amino acid positions 13 and 17 in HLA-DRB1 and 156 in HLA-B. No significant association remains.

Genome-wide significant association was observed for multiple SNPs across the MHC on chromosome 6p21.3 (Figs 1 and 2). Considering the MHC region in more detail, an analysis of SNPs, imputed HLA alleles and amino-acid constituents of HLA alleles was performed. We also directly compared imputation of HLA alleles from the GWAS data to high resolution HLA genotypes, and found strong concordance between imputed two-digit HLA types and variants (97.6%-99.4%), and slightly lower concordance for four-digit resolution (95.8–98.7%; Table 2).

**Table 2:** Accuracy of HLA typing by imputation compared with directly genotyped findings at two–and four-digit resolution.

	*HLA-B*	*HLA-C*	*HLA-DRB1*	*HLA-DQB1*
Two-digit resolution	99.4	98.9	97.6	98.7
Four-digit resolution	98.7	98.2	96.7	95.8

The strongest associated SNP, rs9271858 (OR = 7.44, *P =* 5.20 × 10^−15^), lies in the MHC Class II region near HLA-DQA1*0102. This SNP is in strong linkage disequilibrium with rs9272143 (r^2^ = 1). Conditioning this signal for the previously reported *MICA5*.*1* and *HLA-DRB1*-eQTL associations at rs67841474 and rs9272143, residual MHC association remained (HLA-B*0702, OR = 1.22 *P =* 2.39 × 10^−5^; HLA-B*1501, OR = 0.62 *P =* 1.11 × 10^−9^; HLA-DQB1*0602, OR = 1.29 *P =* 2.49 × 10^−6^; HLA-DRB1*1501, OR = 1.28 *P =* 8.61 × 10^−6^).

### HLA risk alleles

**Fig 3:**
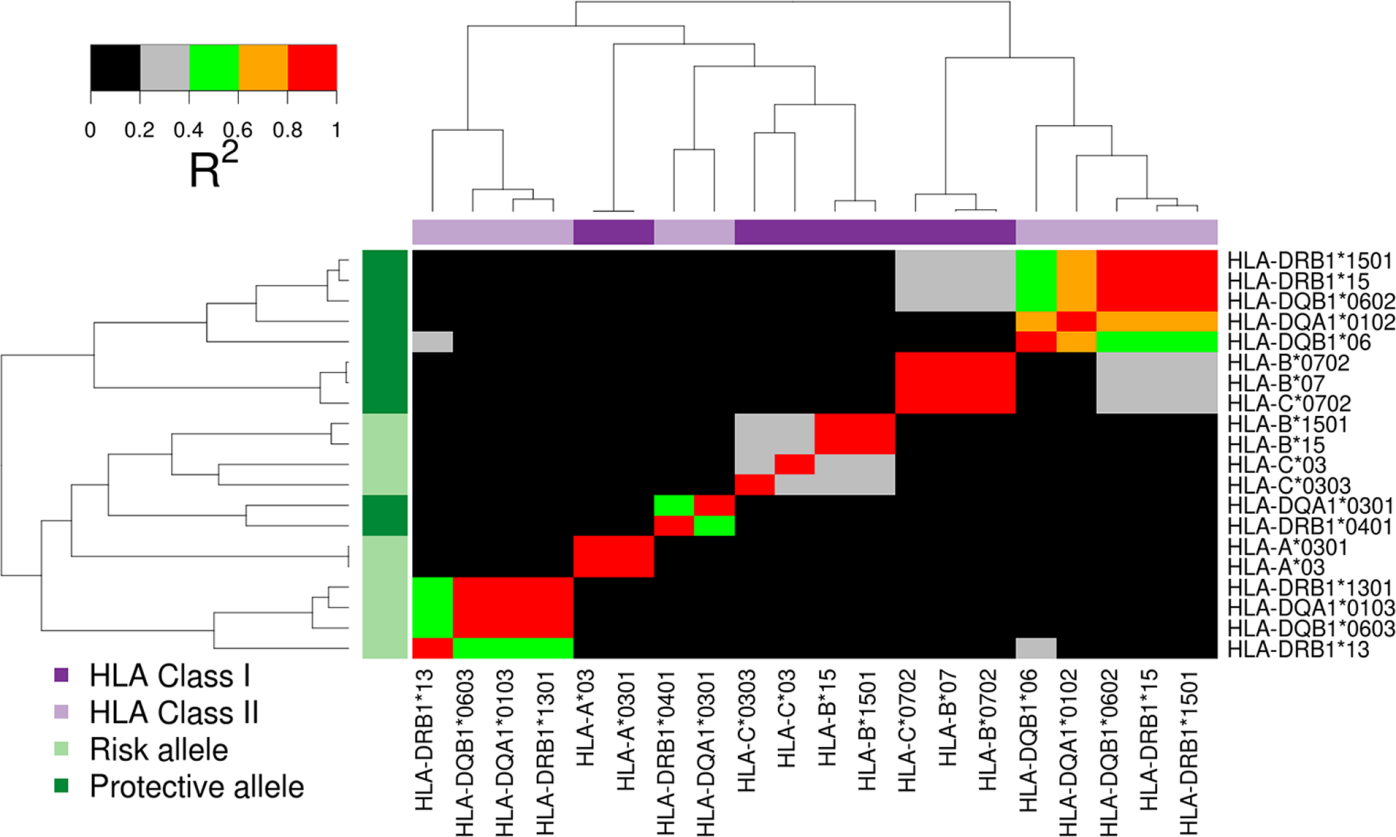
Pairwise linkage disequilibrium (*r*^2^) plot of HLA alleles associated with cervical cancer. HLA alleles have been clustered according to their pairwise linkage disequilibrium on both the x- and y-axes. On the left-hand y-axis they are labelled as to whether they are risk or protective alleles in the overall cervical cancer dataset, and on the top x-axis according to whether they are HLA Class I or II alleles.

Our analysis identifies two independent risk HLA-haplotypes, HLA-DRB1*15/HLA-DQB1*0602/HLA-DQA1*0102 and HLA-DRB1*0401/HLA-DQA1*0301. Within each haplotype the HLA alleles are in strong LD (Fig 3), whereby conditioning on any one of the HLA alleles controls for the association signal at the other alleles.

The strongest risk HLA-associations are seen with HLA-DQB1*0602 (OR = 1.44, *P =* 4.46 × 10^−12^, Table 3) and HLA-DRB1*1501 (OR = 1.43, *P =* 5.55 × 10^−12^). HLA-DQB1*0602 is in positive linkage disequilibrium with the HLA Class II risk alleles HLA-DRB1*1501 (r^2^ = 0.93) and HLA-DQA1*0102 (r^2^ = 0.64), and in lower linkage disequilibrium with the HLA Class I risk alleles HLA-C*0702 (r^2^ = 0.25) and HLA-B*0702 (r^2^ = 0.28; Fig 3). Controlling for the association at HLA-DQB1*0602 or HLA-DRB1*1501 controls for the association at each of the other HLA Class I or II alleles in these linkage disequilibrium blocks (*P >* 0.05), whereas controlling for either or both of HLA-B*0702 or HLA-C*0702 leaves strong residual association at both HLA-DQB1*0602 and HLA-DRB1*1501 (OR>1.3 *P<*1.5 × 10^−5^ all analyses). This indicates that the primary association of this haplotype is best tagged by HLA-DRB1*1501/HLA-DQB1*0602/HLA-DQA1*0102, and that the associations of HLA-B*0702/HLA-C*0702 are likely to be due to linkage disequilibrium rather than themselves being disease-causative.

**Table 3:** Conditional logistic regression analysis of imputed HLA alleles for the overall dataset for alleles scoring *P<*10^−5^ in either the primary analysis or after conditioning on stated variants.

			Conditioned *P-*values						
HLA Allele	FRQ	Odds ratio	Unconditioned *P-*values	DQB1*0602	DQB1*0603	B*15	B*15 /DQB1*0602/DQB1*0603	DRB1*0401	rs2596560
C*03	0.153	0.82	4.03 × 10^−5^	0.00089	0.00022	0.32	0.61	1.03 × 10^−6^	0.16
C*0303	0.055	0.66	9.28 × 10^−7^	5.20 × 10^−6^	4.53 × 10^−5^	0.026	0.088	9.77 × 10^−8^	0.013
C*0702	0.170	1.26	2.40 × 10^−7^	0.082	1.19 × 10^−6^	1.14 × 10^−5^	0.22	4.84 × 10^−8^	0.51
B*07	0.162	1.32	1.90 × 10^−9^	0.011	1.30 × 10^−8^	1.78 × 10^−7^	0.041	2.72 × 10^−10^	0.15
B*0702	0.16	1.31	3.86 × 10^−9^	0.017	2.48 × 10^−8^	3.22 × 10^−7^	0.057	5.63 × 10^−10^	0.19
B*15	0.072	0.64	1.56 × 10^−9^	2.87 × 10^−8^	1.17 × 10^−7^	NA	1	1.17 × 10^−13^	0.028
B*1501	0.065	0.63	4.44 × 10^−9^	7.18 × 10^−8^	3.13 × 10^−7^	0.97	0.87	2.71 × 10^−13^	0.028
DRB1*04	0.206	1.17	0.00018	2.95 × 10^−8^	0.0028	1.50 × 10^−6^	7.35 × 10^−9^	0.28	0.12
DRB1*0401	0.119	1.24	7.13 × 10^−5^	1.00 × 10^−7^	0.00072	1.52 × 10^−8^	3.94 × 10^−10^	1	0.33
DRB1*13	0.106	0.69	1.13 × 10^−9^	2.96 × 10^−7^	0.0017	2.46 × 10^−8^	0.012	9.49 × 10^−9^	0.77
DRB1*1301	0.055	0.62	2.87 × 10^−8^	9.95 × 10^−7^	0.35	2.81 × 10^−6^	0.85	7.91 × 10^−8^	0.46
DRB1*15	0.153	1.42	1.40 × 10^−11^	0.99	4.73 × 10^−10^	4.58 × 10^−10^	0.94	2.62 × 10^−14^	0.062
DRB1*1501	0.14	1.43	5.55 × 10^−12^	NA	1.75 × 10^−10^	1.79 × 10^−10^	0.68	1.32 × 10^−14^	0.57
DQA1*01	0.402	0.97	0.40	2.28 × 10^−7^	0.00081	0.36	0.00036	0.52	0.20
DQA1*0102	0.205	1.25	2.59 × 10^−6^	0.066	2.33 × 10^−5^	3.30 × 10^−5^	0.037	9.56 × 10^−9^	0.15
DQA1*0103	0.061	0.63	3.36 × 10^−8^	1.33 × 10^−6^	0.35	2.54 × 10^−6^	0.59	1.17 × 10^−7^	0.35
DQA1*03	0.224	1.16	0.00027	3.51 × 10^−8^	0.0046	2.30 × 10^−6^	0.59	1.17 × 10^−7^	0.13
DQA1*0301	0.224	1.16	0.00027	3.51 × 10^−8^	0.0046	2.30 × 10^−6^	1.04 × 10^−8^	0.31	0.13
DQB1*03	0.344	1.08	0.051	1.56 × 10^−5^	0.41	0.0054	5.08 × 10^−5^	0.62	0.70
DQB1*0302	0.129	1.08	0.14	0.0068	0.37	0.0023	0.00046	0.68	0.40
DQB1*06	0.246	1.04	0.32	2.12 × 10^−7^	5.89 × 10^−5^	0.26	0.00046	0.68	0.60
DQB1*0602	0.141	1.44	4.46 × 10^−12^	NA	1.93 × 10^−10^	1.46 × 10^−10^	0.011	0.041	0.30
DQB1*0603	0.057	0.63	4.17 × 10^−8^	1.00 × 10^−6^	NA	3.65 × 10^−6^	1	1.16 × 10^−14^	0.36

FRQ, allele frequency in controls; NA, not analysed, as conditioned on.

Moving along the x axis of Fig 3, the second block of risk associations are seen at HLA-DRB1*0401/HLA-DQA1*0301. In the uncontrolled analysis, nominal associations were observed for each allele in Table 3 (HLA-DQA1*0301 (OR = 1.16, *P =* 2.80 × 10^−4^), HLA-DRB1*0401 (OR = 1.24, *P =* 7.98 × 10^−5^)). Conditioning on the HLA-DQB1*0602 risk linkage disequilibrium block strengthens these associations (HLA-DQA1*0301 to OR = 1.27, *P =* 2.4 × 10^−7^ and HLA-DRB1*0401 to OR = 1.25, *P =* 4.7 × 10^−7^). If both risk *and* protective alleles are used to condition (i.e., additionally including HLA-DQB1*0603 and HLA-B*15), the residual association with HLA-DQA1*0301 and HLA-DRB1*0401 becomes stronger still (both at *P<*1 × 10^−8^). These two alleles are in moderate linkage disequilibrium with one another (r^2^ = 0.49), but not with HLA-DQB1*0602, HLA-DQB1*0603 or HLA-B*15 (r^2^<0.05, all comparisons). Controlling for either HLA-DRB1*0401 or HLA-DQA1*0301 controls for association at the other allele (residual association *P >* 0.05), but significant associations remain at HLA-DQB1*0602, and HLA-B*15 (residual associations OR>1.5 *P<*5 × 10^−15^). Controlling for HLA-DRB1*0401, HLA-DQB1*0602, HLA-DQB1*0603, and HLA-B*15, no HLA allele or MHC SNP shows any association (*P >* 0.005). This indicates that HLA-DQA1*0301 and HLA-DRB1*0401 tag a further independent association for cervical neoplasia, and that no further major risk MHC associations remain.

### HLA protective alleles

The strongest protective association was seen with HLA-B*15 (OR = 0.64, *P =* 1.56 × 10^−9^), driven primarily by the HLA-B*1501 allele which makes up 90% of HLA-B*15 alleles in this dataset and is itself strongly associated with cervical neoplasia (OR = 0.63, *P =* 4.44 × 10^−9^). HLA-C*0303 which is in moderate LD with HLA-B*15 (r^2^ = 0.27) also shows protective association (OR = 0.66, *P =* 9.28 × 10^−7^). Conditioning on HLA-B*15 completely controls for the association at HLA-C*0303 (OR = 0.80, *P =* 0.03), whereas conditioning on HLA-C*0303 leaves residual association at HLA-B*15 (OR = 0.7 *P =* 2.04 × 10^−5^), implying that the causative association is with HLA-B*15.

Reduced risk is also observed with the HLA Class II alleles HLA-DRB1*13 (OR = 0.62, *P =* 2.87 × 10^−8^), HLA-DQB1*0603 (OR = 0.63, *P =* 4.17 × 10^−8^), and HLA-DQA1*0103 (OR = 0.63, *P =* 3.36 × 10^−8^). These three HLA Class II alleles are in strong positive linkage disequilibrium with one another, but not with the protective HLA Class I alleles (Fig 3). Controlling for the association at HLA-DQB1*0603, no residual association is seen at HLA-DQA1*0103 (OR = 0.77 *P =* 0.35), and only minor association is seen at HLA-DRB1*13 (OR = 0.77 *P =* 0.002), but residual association remains at HLA-B*15 (OR = 0.68 *P =* 1.17 × 10^−7^) and HLA-C*0303 (OR = 0.71 *P =* 4.53 × 10^−5^). This indicates that there are separate protective associations with the HLA Class II haplotype HLA-DRB1*13/HLA-DQB1*0603/HLA-DQA1*0103, and the HLA Class I allele HLA-B*15, and that other protective allelic associations are likely to be due to linkage disequilibrium with these associated variants.

### Amino-acid associations

In unconditional analyses, associations with *P<*10^−6^ are observed with HLA-DRB1 amino acid positions -25, -16, -1, 11, 12, 13, 32, 70, 71, 96, 133, 142, and 149 (relative to the reference HLA-DRB1 sequence). At HLA-DQB1, associations at *P<*10^−6^ are seen with amino acid position 9 and 30, and at HLA-DQA1 at amino acid positions 24, 41, and 130.

The strongest association observed in all analyses of SNPs, amino-acids and HLA types was with amino-acid position 71 in HLA-DRB1 (with possible amino acids K, A, E, R, KA, KE, or KR, *P =* 1.25 × 10^−17^; Table 4, Fig 2A). The amino-acids at this locus have a gradient of association with cervical neoplasia risk, with the presence of alanine being associated with increased risk of cervical neoplasia (OR = 1.42, *P =* 1.44 × 10^−11^), and of glutamic acid with reduced risk of the disease (OR = 0.67, *P =* 2.57 × 10^−11^; S1 Table). Controlling for the association with HLA-DRB1 amino acid 71 controls for the association (*P >* 0.0005) with all the HLA Class II alleles except HLA-DRB1*0401 (*P =* 3.29 × 10^−4^) and HLA-DQA1*0501 (*P =* 4.17 × 10^−4^; Table 4). Similarly, controlling for amino-acid position 13 in HLA-DRB1 controls for all associations at imputed HLA Class II amino acids (conditioned *P >* 10^−3^), with the exceptions of HLA-DQA1*0103 (conditioned *P =* 9.32 × 10^−5^) and HLA-DRB1*13 (conditioned *P =* 2.54 × 10^−4^; Table 4). Conditioning on both HLA-DRB1 amino acid 13 and 71 controls for all HLA Class II (but not Class I) allele and amino acid associations (*P >* 0.03 for all HLA Class II alleles and amino acids; Fig 2B). This suggests that these two amino-acids are of functional importance in the mechanism by which HLA Class II alleles influence cervical neoplasia risk. The side chains of these amino acids are part of pocket 4 of HLA-DRB1, which is defined by positions 9, 13, 70, 71, 74 and 78. Serine (position 13) and glutamic acid (position 71) are associated with reduced risk of cervical neoplasia, whilst histidine/arginine (position 13) and alanine (position 71) are associated with increased risk (S2 Table).

**Table 4:** Conditional logistic regression analysis of imputed HLA amino acids at HLA-B position 156 (B_156), HLA-DRB1 position 13 (DRB1_13) and 71 (DRB1_71).

		Conditioned *P-*values				
HLA Allele	Unconditioned *P-*values	DRB1_13	DRB1_71	DRB1_13, DRB1_71	B_156	DRB1_13, DRB1_71, B_156
B_156	9.97 × 10^−16^	8.20 × 10^−12^	1.078 × 10^−7^	9.82 × 10^−10^	1	1
DRB1_13	5.20 × 10^−17^	1	0.0066	1	2.30 × 10^−13^	1
DRB1_71	1.25 × 10^−17^	0.00059	1	1	1.12 × 10^−9^	1
C*03	4.03 × 10^−5^	1.016 × 10^−5^	0.0089	0.00022	0.47	0.71
C*0303	9.28 × 10^−7^	5.26 × 10^−7^	4.27 × 10^−5^	5.27 × 10^−6^	0.11	0.15
C*0702	2.40 × 10^−7^	0.16	0.067	0.12	0.12	0.097
B*07	1.90 × 10^−9^	0.028	0.0077	0.018	0.65	0.68
B*0702	3.86 × 10^−9^	0.040	0.012	0.026	0.53	0.43
B*15	1.56 × 10^−9^	1.24 × 10^−10^	7.63 × 10^−8^	7.97 × 10^−10^	0.28	0.31
B*1501	4.44 × 10^−9^	3.23 × 10^−10^	1.36 × 10^−7^	1.58 × 10^−9^	0.69	0.65
DRB1*04	0.00018	0.74	0.00061	0.83	4.09 × 10^−7^	0.74
DRB1*0401	7.13 × 10^−5^	0.062	0.00033	0.16	2.27 × 10^−8^	0.013
DRB1*13	1.13 × 10^−9^	0.00077	0.77	0.92	4.74 × 10^−7^	0.62
DRB1*1301	2.87 × 10^−8^	0.00025	0.14	0.15	7.50 × 10^−6^	0.52
DRB1*15	1.40 × 10^−11^	0.75	0.097	0.082	3.97 × 10^−5^	0.029
DRB1*1501	5.55 × 10^−12^	0.29	0.15	0.19	2.48 × 10^−5^	0.31
DQA1*01	0.40	0.0044	0.16	0.50	0.033	0.33
DQA1*0102	2.59 × 10^−6^	0.79	0.028	0.036	0.027	0.12
DQA1*0103	3.36 × 10^−8^	9.32 × 10^−5^	0.057	0.065	9.18 × 10^−6^	0.30
DQA1*03	0.00027	0.62	0.00093	0.73	4.68 × 10^−7^	0.54
DQA1*05	0.0058	0.024	0.00042	0.092	0.00008	0.025
DQA1*0301	0.00027	0.62	0.00093	0.73	4.68 × 10^−7^	0.54
DQB1*03	0.051	0.90	0.038	0.59	0.00079	0.78
DQB1*0302	0.14	0.018	0.31	0.036	0.00053	0.66
DQB1*06	0.32	0.0013	0.78	0.73	0.43	0.45
DQB1*0602	4.46 × 10^−12^	0.22	0.094	0.13	2.097 × 10^−5^	0.23
DQB1*0603	4.17 × 10^−8^	0.00020	0.11	0.12	8.018 × 10^−6^	0.45

Genome-wide significant HLA Class I association remains after conditioning on HLA-DRB1 amino acids 13 and 71, with the strongest associated allele being HLA-B*15, for which strong residual association is seen (*P =* 7.97 × 10^−10^). The strongest amino-acid association in this analysis is with the amino-acid 156 in HLA-B (*P =* 9.82 × 10^−10^). Controlling for the association of this amino acid, only minor residual HLA or MHC SNP association is observed (*P >* 0.0005; Table 4, Fig 2C).

### Associations with cervical cancer subtypes

#### Squamous cell carcinoma

Among the cases, 871 were known to have high-grade squamous cell lesions or squamous cell carcinoma and 546 had in situ adenocarcinoma or adenocarcinoma. Association findings for classical HLA loci for each histopathological type are presented in Table 5. For squamous cell carcinoma, the strongest risk HLA allele associations were seen with HLA-DQB1*0602 (OR = 1.52, *P =* 1.47 × 10^−7^) and HLA-DRB1*1501 (OR = 1.50, *P =* 2.95 × 10^−7^). Controlling for these associations, no further HLA associations were observed.

**Table 5:** HLA associations with cervical cancer histopathological type, and HPV genotype, for alleles scoring *P<*0.005 in at least one sub-analysis.

			Squamous Cell Carcinoma		Adenocarcinoma		HPV16		HPV18	
HLA Allele	BP	FRQ	OR	*P-*VALUE	OR	*P-*VALUE	OR	*P-*VALUE	OR	*P-*VALUE
A*03	30019970	0.1604	1.24	0.01	1.37	0.0031	1.25	0.022	1.16	0.41
A*0301	30019970	0.167	1.24	0.01	1.38	0.0028	1.25	0.021	1.16	0.41
C*03	31346171	0.1552	0.77	0.01	0.81	0.05	0.79	0.015	0.93	0.67
C*0303	31346171	0.0588	0.65	0.003	0.74	0.093	0.63	0.0076	0.58	0.098
C*0702	31346171	0.1641	1.20	0.01	1.4	2.10 × 10^−4^	1.3	0.0011	1.43	0.016
B*07	31431272	0.1542	1.27	8.61 × 10^−4^	1.48	2.80 × 10^−5^	1.43	1.58 × 10^−5^	1.44	0.016
B*0702	31431272	0.153	1.26	0.002	1.48	2.65 × 10^−5^	1.44	1.1 × 10^−5^	1.45	0.014
B*15	31431272	0.0772	0.55	4.77 × 10^−6^	0.732	0.039	0.47	4.24 × 10^−6^	1	0.95
B*1501	31431272	0.0696	0.53	5.61 × 10^−6^	0.72	0.043	0.47	1.61 × 10^−5^	0.99	0.96
B*39	31431272	0.0155	0.43	0.009	0.64	0.21	0.3	0.0031	1.26	0.61
DRB1*13	32660042	0.1108	0.71	5.27 × 10^−4^	0.54	1.83 × 10^−5^	0.7	0.0022	0.51	0.0058
DRB1*1301	32660042	0.0588	0.69	0.009	0.67	0.042	0.68	0.026	0.39	0.017
DRB1*1302	32660042	0.0445	0.71	0.03	0.42	3.91 × 10^−4^	0.68	0.031	0.73	0.34
DRB1*15	32660042	0.145	1.47	8.75 × 10^−7^	1.49	1.17 × 10^−4^	1.5	1.07 × 10^−5^	1.68	0.0013
DRB1*1501	32660042	0.1371	1.50	2.95 × 10^−7^	1.46	3.23 × 10^−4^	1.49	1.55 × 10^−5^	1.65	0.0025
DQA1*0102	32716284	0.1968	1.29	5.37 × 10^−4^	1.09	0.36	1.23	0.016	1.44	0.016
DQA1*0103	32716284	0.0649	0.70	0.007	0.76	0.12	0.74	0.056	0.5	0.041
DQA1*05	32716284	0.2247	0.92	0.27	0.84	0.076	0.7	8.72 × 10^−5^	0.92	0.58
DQA1*0501	32716284	0.2247	0.92	0.27	0.84	0.076	0.7	8.72 × 10^−5^	0.92	0.58
DQB1*0602	32739039	0.1326	1.52	1.58 × 10^−7^	1.48	2.60 × 10^−4^	1.5	1.93 × 10^−5^	1.65	0.003
DQB1*0603	32739039	0.0613	0.69	0.008	0.66	0.032	0.68	0.025	0.42	0.021
DPB1*0401	33157346	0.4268	1.15	0.03	1.29	0.0019	1.1	0.17	1.26	0.079

BP, base-pairs from the chromosome 6 p-terminus; FRQ, allele frequency in control; OR, odds ratio. The number of squamous cell cancer cases is 871, adenocarcinomas 546, HPV16-associated cancers 668 and HPV18-associated cancers 166.

A reduced risk of squamous cell carcinoma was seen with both HLA Class I (HLA-B*15 (OR = 0.55, *P =* 4.78 × 10^−6^)) and HLA Class II alleles (HLA-DQA1*0103 (OR = 0.69, *P =* 0.006), HLA-DRB1*13 (OR = 0.71, *P =* 5.27 × 10^−4^), HLA-DQB1*0603 (OR = 0.69, *P =* 0.007)). HLA-DQB1*0603 and HLA-DRB1*13, are, as mentioned above, in tight positive linkage disequilibrium. Conditioning on HLA-DQB1*0603 left no residual association observed with HLA-DRB1*13 (OR = 0.73 *P =* 0.03), although significant associations with reduced risk remained at HLA-B*15 (OR = 1.44, *P =* 4.22 × 10^−5^). HLA-B*15 is not in linkage disequilibrium with the HLA Class II risk alleles HLA-DQA1*0103, HLA-DRB1*13, or HLA-DQB1*0603 (r^2^<0.05 for each). This analysis thus demonstrates the existence of separate HLA Class I and Class II haplotypes associated with reduced risk of squamous cell carcinoma. Finally, controlling for the amino acid positions 13 and 71 in HLA-DRB1, and position 156 in HLA-B, no residual HLA allele or amino acid associations were observed (*P >* 0.001).

#### Adenocarcinoma

As with squamous cell carcinoma, risk associations were seen between adenocarcinoma and HLA Class I (HLA-B*0702 (OR = 1.48, *P =* 2.65 × 10^−5^)) and Class II (HLA-DRB1*15 (OR = 1.49, *P =* 0.00012; HLA-DQB1*0602 (OR = 1.48, *P =* 0.00026) alleles. Controlling for the association at any one of these alleles, no residual association was seen at the remaining risk alleles (*P >* 0.01).

A strongly reduced risk of adenocarcinoma was seen with HLA-DRB1*13 (OR = 0.54, *P =* 1.83 × 10^−5^). Reduced risk was also seen in association with HLA-DQB1*0603 (OR 0.66, *P =* 0.032), HLA-B*15 (OR = 0.73, *P =* 0.039) and HLA-C*3 (OR = 0.81, *P =* 0.050). Conditional analysis again indicated that the HLA Class I and II associations were separate, as was the case with overall HPV cervical neoplasia and squamous cell carcinoma risk. Thus, controlling for the amino acid positions 13 and 71 in HLA-DRB1, and position 156 in HLA-B, no residual HLA allele or amino acid associations are observed with adenocarcinoma (*P >* 0.001).

#### HPV genotypes

HLA alleles were associated with disease when assessed via the HPV genotype detected in the cervix, without regard to the histologic classification of the tumor. Comparing HPV16 cervical cancer cases (n = 652) with healthy controls (n = 6419), similar risk associations were seen as with cervical neoplasia overall. The HLA Class I risk was most strongly associated with HLA-B*0702 (OR = 1.44, *P =* 1.10 × 10^−5^), and HLA Class II was associated with HLA-DRB1*15 (OR = 1.50, *P =* 1.07 × 10^−5^); likewise, reduced risk was seen most strongly with HLA-B*15 (OR = 0.47, *P =* 4.24 × 10^−6^).

Strong associations between HPV16 cervical cancer cases and amino acid positions 13 and 71 were found in HLA-DRB1 (*P =* 2.69 × 10^−13^ and 6.22 × 10^−7^ respectively), and position 156 in HLA-B (*P =* 4.93 × 10^−8^). Controlling for amino acids 13 and 71 in HLA-DRB1 left no residual HLA Class II allele or amino acid associations with HPV16-associated cervical cancer (*P >* 0.001). Conditioning on these amino acids and position 156 in HLA-B, some residual HLA Class I association was seen with amino acid position 67, although this was much less significant that in the unconditioned analysis (unconditioned *P =* 4.41 × 10^−8^; conditioned *P =* 2.89 × 10^−4^).

Considering HPV18-associated cervical cancer (n = 166 cases), association with increased risk was strongest with HLA-DRB1*15 (OR = 1.68, *P =* 0.0013) and HLA-DQB1*0602 (OR = 1.65, *P =* 0.0030), with only nominal HLA Class I risk associations seen with HLA-B*0702 and HLA-C*0702.

In contrast to the strong reduction in risk of HPV16-associated cervical cancer seen in HLA-B*15 carriers, no association was seen with this allele and HPV18-associated cervical cancer (OR = 1.00, *P =* 0.99; comparing HLA-B*15 counts in HPV16 and HPV18-associated cancers OR = 0.53, *P =* 0.013). Reduced risk of HPV18-associated cervical cancer was seen with HLA Class II alleles (HLA-DRB1*13 (OR = 0.51, *P =* 0.0058), HLA-DQB1*0603 (OR = 0.42, *P =* 0.021) and HLA-DQA1*0103 (OR = 0.50, *P =* 0.041)).

Considering amino acid associations of HPV18-associated cervical cancer, association was again seen with amino acid position 13 in HLA-DRB1 (*P =* 7.99 × 10^−5^), but only modest association with amino acid position 71 in HLA-DRB1 (*P =* 0.0019). Controlling for the association of these two amino-acids, association is observed with HLA-DPB1*0701 (*P =* 1.78 × 10^−5^), but no other residual HLA Class II allele or amino acid associations seen (*P >* 0.005). HLA-DPB1*0701 shows no association in any other analysis in this study, and may represent an artefact of the small sample size available for HPV18 analyses (n = 166). Consistent with the weak HLA-B*15 association with HPV18-associated cervical cancer, only marginal association was seen in this group with position 156 in HLA-B (*P =* 0.014).

#### Replication and non-MHC findings

Of the two loci previously reported to be associated with cervical cancer in Chinese (*EXOC1* and *GSDMB*), no association was seen in the current study (S3 Table, S2 Fig and S3 Fig). Considering other SNPs previously reported to be associated with cervical cancer, only at the MHC *TNF* locus was association observed in the current study (S3 Table, S4 Fig); as stated above this association is not observed having conditioned on the major HLA protective and risk alleles or amino-acids. Association was seen with the lowest *P-*value SNPs at the loci *OAS3*, *EVER1/2*, and *IL12RB1* (5 × 10^−5^ < *P<*0.05), where the current study did not have findings either for the previously reported SNP or a close proxy (r^2^>0.9; S3 Table). Suggestive association was observed with SNPs at 9 non-MHC loci (see S4 Table and S5–S13 Figs). No association was seen with non-MHC SNPs associated with oral and pharyngeal cancer [28].

#### Heritability and genetic risk prediction

Common-variant heritability for cervical neoplasia was assessed by both the observed scale and the liability threshold method. The observed scale estimate was 0.56 (SE = 0.037). This method makes the assumption of multivariate normality, and thus can be biased by the presence of a large major gene effect. The liability threshold method requires the population prevalence of the disease or trait being studied to be known. The population prevalence of persistent HPV infection is not well defined but thought to be about 1% [2], giving an estimated common variant heritability of 0.36 (SE = 0.024). Controlling for the most strongly associated MHC SNP, rs9271858, did not change these estimates, and calculating the heritability using all chromosomes except chromosome 6 gave similar results (heritability of 0.33). Removing CIN2 cases did not affect this estimate.

**Fig 4:**
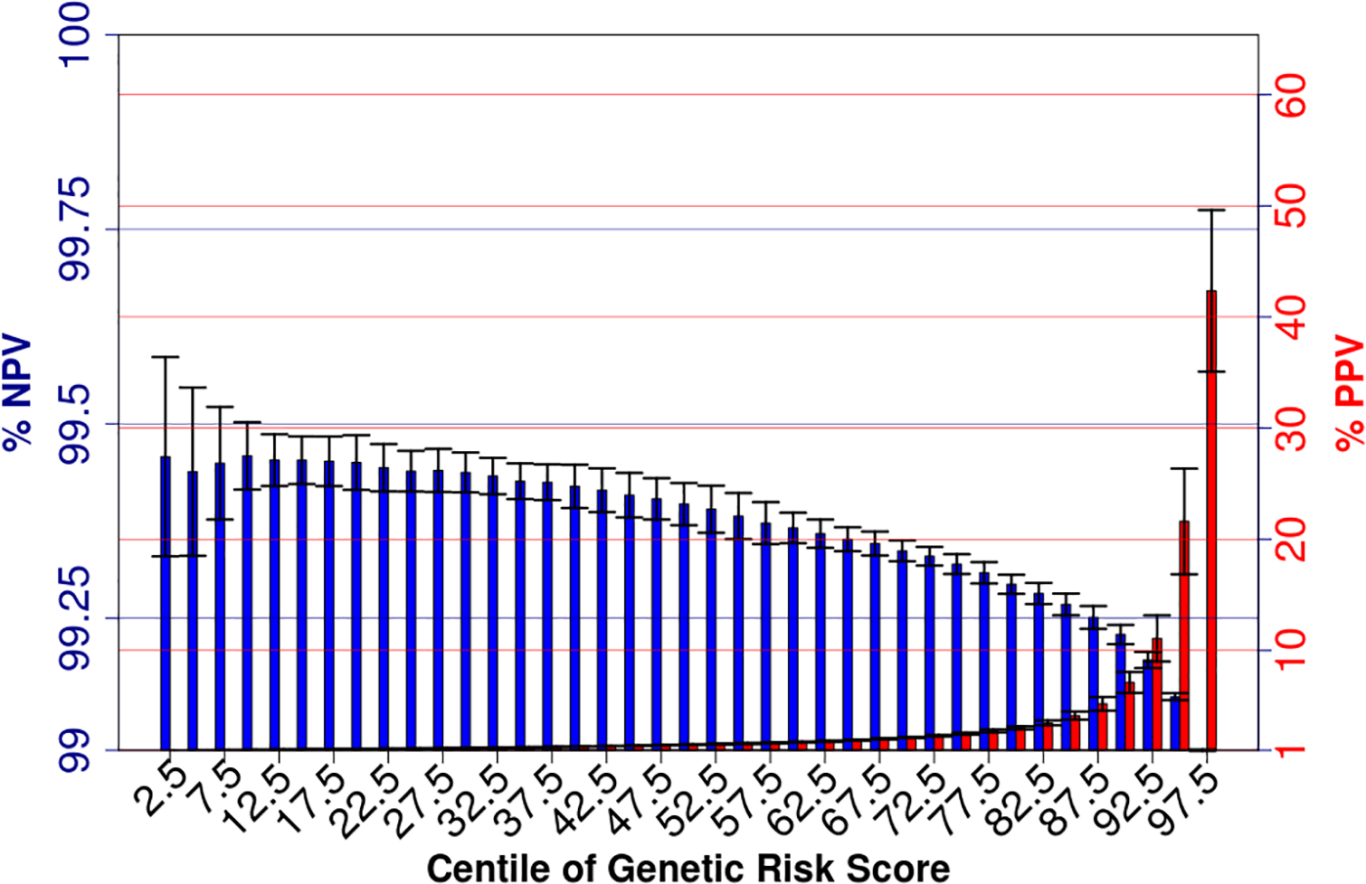
Positive and negative predictive values for cervical neoplasia for centiles of genetic risk scores. Error Bars denote 2 standard deviations based on 10-fold cross validation.

Considering genetic risk prediction, using loci with *P >* 10^-4^, the discriminatory capacity of genetic risk score was moderate (maximum area under the curve (AUC) = 0.68)). Assuming a 1% risk of developing cervical neoplasia amongst the population screened (i.e. amongst HPV-infected women), the negative predictive value for women in the lower lower 50% of genetic risk is 99.40% (SD = 0.03%; i.e. <0.6% chance of developing cervical neoplasia). Those in the top 10% of genetic risk had an estimated genetic risk of developing cervical neoplasia of 7.1% (SD = 0.009%), and those in the top 5% had a risk of developing cervical neoplasia of 22% (SD = 5%). Positive and negative predictive values are given for different centiles in Fig 4.

## Discussion

This study demonstrates that host genetic variants are major determinants of HPV-associated cervical neoplasia. It confirms the strong association of the MHC with cervical neoplasia, and specifically identifies the amino acid positions within both HLA Class I and Class II alleles. These findings are consistent with roles for both CD4 and CD8 T-lymphocytes in disease pathogenesis, given the known role of these genes in presentation of antigen to these cell types. Both risk and protective associations were seen, providing evidence that some alleles have greater roles in relation to particular HPV genotypes and histological subtypes. Furthermore, these findings at least partially explain the differential association of HPV16 and HPV18 with cervical squamous cell carcinoma compared with adenocarcinoma.

Overall, three haplotypes, HLA-DRB1*15/HLA-DQB1*0602/HLA-DQA1*0102, HLA-B*0702/HLA-C*0702, and HLA-DRB1*0401/HLA-DQA1*0301, were associated with increased risk of both HPV16 and HPV18-associated cervical cancer, and for the development of both squamous cell carcinoma and adenocarcinoma. Conditional analysis indicated that risk was primarily driven by the HLA Class II alleles HLA-DRB1*1501/HLA-DQB1*0602/HLA-DQA1*0102, with the HLA Class I associations with HLA-B*0702/HLA-C*0702 being due to linkage disequilibrium. The haplotype HLA-DQA1*0301/HLA-DRB1*0401 was independently associated with increased disease risk though with a smaller effect size compared with the HLA-DRB1*1501/HLA-DQB1*0602/HLA-DQA1*0102 haplotype. Perhaps because of lower power due to its smaller effect size, no association was observed between the HLA-DQA1*0301 or HLA-DRB1*0401 and specific cervical cancer histologic types or HPV DNA types.

An independent HLA Class I haplotype tagged primarily by HLA-B*15 was strongly associated with reduced risk of squamous cell carcinoma and HPV16-associated cervical cancer but had only marginal association with adenocarcinoma (*P =* 0.039) and no association with HPV18-associated cervical cancer (*P =* 0.95). HPV18 is more prevalent in adenocarcinoma than in squamous cell cancers, and this result whilst limited in power raises the possibility that the difference in HPV type distribution between histologic types is partly due to host genetic factors rather than purely arising from differences in tissue tropism and pathogenicity of HPV16 and HPV18. Recent cervical cancer sequencing studies have demonstrated a high carriage rate of deletions involving HLA-B providing further evidence that HLA-B is directly involved in cervical cancer risk or pathogenesis [29]. Once mutational profiles of sufficient tumours of different histological type are reported, it will be interesting to use this data to test the hypothesis that *HLA-B* mutations differentially predispose to different histological types of cervical cancer.

In addition to the strong reduced risk of cervical neoplasia associated with HLA-B*15, the haplotype HLA-DRB1*1301/HLA-DQB1*0603 (and in some analyses HLA-DQA1*0103) was associated with reduced risk of squamous cell cancer, adenocarcinoma, and HPV16- and HPV18-related cervical neoplasia. The HLA-DRB1*1301/HLA-DQA1*0103/HLA-DQB1*0603 haplotype has previously been associated with protection from oral and oropharyngeal cancer, particularly in HPV-positive cases (OR = 0.23, *P =* 1.6 × 10^−6^) [28]. This is of particularly interest given that HPV16 is the most common HPV genotype associated with oropharyngeal cancer.

To further assess the signals from the HLA region, we examined risk of cervical neoplasia at the amino acid level. We observed that the HLA Class II haplotype associations are driven by carriage of particular amino acids at HLA-DRB1 positions 13 and 71. The main risk haplotype, HLA-DRB1*1501/HLA-DQB1*0602/HLA-DQA1*0102, carries the risk amino-acid alanine on HLA-DRB1*1501 at HLA-DRB1 position 71 and the risk amino acid arginine at position 13. The secondary risk haplotype HLA-DRB1*0401/HLA-DQA1*0301 carries the risk amino-acid histidine on HLA-DRB1*0401 at HLA-DRB1 position 13, but at position 71 carries the amino acid lysine which is of neutral effect (see S1 and S2 Figs).

In contrast, the main protective haplotype HLA-DRB1*1301/HLA-DQB1*0603/HLA-DQA1*0103 carries the protective amino-acid glutamic acid on HLA-DRB1*1301 at position 71, and at amino acid position 13 also carries a protective serine. These amino-acids belong to different classes and have varying charges and hydrophobicity. These charge differences at these keys position within pocket 4 of the *HLA-DRB1* the peptide binding groove may interact with putative HPV peptides that are permissive for or protect against development of infections that lead to cervical cancer.

The HLA Class I associations observed are driven by the identity of the amino acid at position 156 in HLA-B. At this position the protective HLA-B*15 allele has tryptophan while other HLA-B alleles have either arginine, leucine or aspartic acid. The amino acid at position 156 in HLA-B is not in the peptide-binding grove, but this particular amino acid position has previously been shown to be associated with persistent viral infection [30]. The classical allele HLA-B*35 has two subtypes HLA-B*3501 and HLA-B*3508 that differ only at amino acid position 156 (HLA-B*3501 leucine, HLA-B*3508 arginine), yet these HLA-B*3501 subtypes have strikingly different peptide affinities to antigens produced by the cytomegalovirus, indicating that amino-acid sequence variation outside of the peptide binding pockets can have significant effects on antiviral immunity [30]. Further studies will be required to determine the relationship between these amino-acid associations and the ability of HLA-DRB1 and HLA-B to present HPV epitopes. Nonetheless, these findings are likely to be of use in design of peptide vaccines for HPV-associated neoplasia.

Previous reports have suggested that the primary MHC associations with cervical neoplasia are with the *MICA5*.*1* allele and an eQTL SNP for *HLA-DRB1* (rs927214), and that the associations of HLA-B*0702 and the HLA-DRB1*1501/HLA-DQB1*0602/HLA-DQA1*0102 haplotype are secondary to the MICA5.1 allele and rs927214 [11]. This study finds no evidence to support these suggestions, with no association observed at either MICA5.1 or rs927214, having controlled for the association of the classical HLA loci. In analyses conditioning on either or both of MICA5.1 and rs927214, residual association was still seen with classical HLA loci, including the HLA-B*0702 and HLA-DRB1*1501/HLA-DQB1*0602/HLA-DQA1*0102 haplotypes. This indicates that the MICA5.1 allele and rs927214 are not primarily associated with cervical neoplasia. It is not clear why the findings from this study are different from those previously reported, and further evaluation in a pooled analysis or in other ethnic groups is warranted.

Our analysis indicates that common variant non-MHC loci contribute 36% of the liability of the disease. Cervical cancer has previously been shown to have significant familiality, indicating that either shared genetic or environmental factors are involved in disease predisposition [3, 31–33]. Twin and family studies have indicated that the heritability of cervical cancer is 22–64% [3, 31, 34, 35]. According to a structural equation modelling study, the heritability of invasive cervical cancer (22%) was higher than that of in situ cancer (13%), while childhood shared environment contributed more to the in situ type (13% vs 3%) [36]. In the current study, removing CIN2 cases did not affect the common genetic variant contribution to disease risk. Unlike these studies, our study design is not subject to shared socio-behavioural or environmental effects within families, which are known to be significant in cervical cancer [3, 35] and may influence heritability estimates. The GCTA method, however, only measures the component of genetic variation related to liability that is captured by the genotypes studied. As this study does not have power to address low frequency or rare genetic variants, the contribution of such variants to cervical cancer liability is not included in our analysis. We demonstrate here that there is substantial but as yet unidentified non-MHC contribution to cervical cancer, suggesting that larger, more powerful, studies are likely to identify further genetic susceptibility factors for this disease.

Consistent with the high heritability of the disease, our analysis shows that genetic risk scoring studies have potential value in identifying women at high risk of the disease. The positive predictive value of the cases with a genetic risk score in the top 10% was 7.1% (SD = 0.93%), and in the top 5% was 21.6% (SD = 4.8%), compared with the risk of cervical neoplasia in HPV carriers of 1%. This suggests that genetic risk screening could be of value in identifying some women with very high risk of developing cervical neoplasia. Women in the lower 50% of genetic risk scores have ≤0.6% (SD = 0.027%) chance of developing cervical cancer, assuming a prevalence of cervical neoplasia of 1% among HPV exposed women. The informativity of low genetic risk scores did not significantly increase in those with more extreme low risk scores, with women in the lower 10% of the genetic risk score distribution having 0.54% (SD = 0.043%) chance of developing cervical cancer. These values may vary depending on the proportion of women who have been HPV infected who progress to cervical neoplasia, which is not well defined in different populations. This suggests that genetic risk scores may have clinical value in determining women at high risk of cervical neoplasia, but not in identifying women at sufficiently low risk to be of clinical value.

In conclusion, this study has demonstrated strong association of MHC haplotypes with increased and reduced risk of HPV-associated cervical cancers, with findings implicating both HLA Class I and Class II loci. These associations are driven by the identity of amino-acids at positions 13 and 71 in HLA-DRB1 and 156 in HLA-B. No non-MHC associations were identified, but strong common variant heritability was demonstrated, indicating that host genetic variation is a major determinant of the likelihood of cervical neoplasia in HPV affected women. Further research is indicated in the potential for genetic risk score analysis in combination with other measures to identify a subset of women at particularly high risk of cervical neoplasia.

## Methods

### Study population

Case and control sets are described in Table 1. Phenotypic information was collected for grade, histology, and HPV genotype where available from the contributing studies. Cancers were histologically classified as either adenocarcinoma, squamous cell carcinoma or other (Table 1). HPV DNA typing performed as part of the original studies was summarized into four groups, those with HPV16 (but not HPV18), those with HPV18 (but not HPV16), those with neither HPV16 nor HPV18, or those carrying both HPV16 and HPV18 (S3 Table). All studies were conducted among majority European descent populations.

### Genotyping

All case samples were genotyped at the University of Queensland Diamantina Institute using Illumina Human660-Quad BeadChips. Controls were either genotyped by the Wellcome Trust Sanger Centre (WTCCC2 cohort) [27], or Erasmus University, Rotterdam (Umea cohort), using Illumina Human610-Quad BeadChips. Bead intensity data were processed and normalized for each sample and genotypes called within participating studies using BeadStudio.

### Statistical methods

Genotype results derived from the two different genotyping chips were combined and the GWAS QC was performed using PLINK [37]. Standard quality control measures were applied including identification and exclusion of cryptic related samples (112 Umea controls, 97 cases), exclusion of samples with an outlying heterozygosity rate (>0.37 or < 0.32) or excess missingness (>5%; 67 Umea controls, 104 cases). SNPs with Hardy-Weinberg equilibrium *P-*values <10^−7^, or minor allele frequencies <5% were excluded. Population stratification was accessed using Shellfish (http://www.stats.ox.ac.uk/~davison/*software*/*shellfish*/*shellfish*.php); after the removal of regions of long range LD the sample set was first spiked with HAPMAP samples to remove ethnic outliers (18 Umea controls, 44 cases), and then the principal components were recalculated using the remaining samples. Four principal components were used as covariates to control for population stratification.

Considering previously associated SNPs from candidate gene or GWAS studies, where the exact SNP was neither genotyped nor imputed in the current study, a proxy SNP with high LD (r^2^>0.9) with the original report was sought. Where no such proxy SNP was available, the SNP with the most significant association at the locus/candidate gene was reported.

The genotype data for SNPs that were common between the datasets were imputed using Impute2 using 1000 Genomes Phase 3 reference data, and association testing performed using SNPTEST [38]. Imputed loci with quality score <0.6 were excluded from the association testing. Detailed investigation of the MHC region and HLA loci was performed using SNP2HLA, an analysis package that performs HLA allele and amino acid imputation from SNP data, and association analysis [39]. HLA amino acid imputation was performed using a reference panel from the Type 1 Diabetes Genetics Consortium (n = 5,225). Loci imputed by SNP2HLA with r^2^<0.5 were excluded and samples where the allele dosage at any HLA type exceeded 2.5 were removed (an additional 25 cases and 67 controls). To assess the accuracy of HLA-allele imputation, previously reported findings from 501 cases that had had *HLA-B*, *-C*, *-DRB1* and *-DQB1* DNA-based direct genotyping performed to four digit resolution in one of the studies included in the GWAS were compared with imputed data [7]. Association and conditional logistic regression analysis of the MHC region was performed using the SNP2HLA dosage files using PLINK and custom R scripts. Study power was calculated using Genetic Power Calculator [40]. The reference sequence for HLA-DRB1 used was GenBank Access number AB829523.1, and for HLA-B was GenBank Accession number AB826450.

Assuming a population prevalence of cervical neoplasia among HPV-infected women of approximately 1% [2] and that the controls were not screened for HPV infection (as was the case in this study), the study has >95% power to detect loci with minor allele frequency = 0.1, D’ = 0.9, with an additive odds ratio of 1.4 or more, at a genome-wide significance threshold of *P<*5 × 10^−8^, or an odds ratio of 1.3 or more at a suggestive significance threshold of *P<*5 × 10^−5^.

The proportion of variance in risk of developing cervical pre-cancer or cancer captured by the SNPs genotyped and imputed in this study was determined using the Genome-wide Complex Trait Analysis (GCTA) method. This uses the available SNP data to assess the degree of relatedness of cases compared with healthy controls to assess heritability in non-familial datasets [41].

Genetic risk scores were calculated for each individual using the adaptive MultiBLUP algorithm [42] using only genotyped SNPs in common between all SNP arrays where the missing rate was less than 5%, the frequency was greater than 2% and the Hardy Weinberg *P-*value for the unaffected individuals was > 1e-7 (n = 277,670 SNPs). A conservative approach was adopted whereby the cohort was divided into independent training and test sets (rather than using a cross-validation approach) so that a training (1341 cases, 3217 controls) and test (3218 controls, 1342 cases) sets was used.The training set was then used to calculate the scoring matrix, which included control of cryptic relatedness using the kinship matrix (near identical results were obtained by using the 4 principal components to control for population stratification in the training and test data). This MultiBLUP algorithm first selects regions based on a *P-*values threshold (option—sig1) obtained using the training cohort. Within these regions all SNPs with a significances threshold greater than a second *P-*value threshold (option—sig2) are considered by the algorithm which then controls for the LD structure. The *P-*value thresholds were optimized by choosing a range of values between 10^−7^ and 5 × 10^−3^ for sig1 and 0.001 and 0.05 for–sig2; the resulting weighted predictors were applied to the test cohort to obtain per sample scores from which the AUC was obtained. Thresholds within these ranges provided AUC ranging from 0.66 to 0.68, with the peak AUC at sig1 = = 5e10^−4^ and sig2 = 0.02 of AUC = 0.68, that included 35 regions and 692 predictors (SNPs), 234 of these within the MHC regions. An example Manhattan plot of the LRT *P-*values for this training set are provided in supplementary figures. Using these final sig1 and sig2 parameters we repeated the training and scoring procedure 10 times using random permutations of samples in the training and test sets to obtain standard deviations (SD) in the predictions. The average AUC was 0.678 (SD = 0.008) with an average of 32 regions (SD = 4) identified in the training examples. Positive and negative predictive values were then calculated using standard methods [43] for all 10 iterations and the mean predictive values and their standard deviat but at position 71 carries the amino acid lysine ion calculated.

## Supporting information

S1 Table Amino-acid associations at amino-acid position 71 in HLA-DRB1. The in phase alleles column denotes which alleles R2 terms refers where P = Present and A = absent; the term P/P denotes that the R2 is with respect to presence of the classical allele and the presence of the amino acids.
(DOCX)

S2 Table Amino-acid associations at amino-acid position 13 in HLA-DRB1. The in phase alleles column denotes which alleles R2 terms refers where P = Present and A = absent; the term P/P denotes that the R2 is with respect to presence of the classical allele and the presence of the amino acids.
(DOCX)

S3 Table GWAS findings at loci previously reported to be associated with cervical cancer. Candidate genes are as reported in original reference.
(DOCX)

S4 Table Non-MHC SNPs achieving suggestive (5 × 10^−8^< *P* <10^−5^) association with cervical neoplasia. (DOCX)

S1 Fig Q-Q plot for overall association findings. Findings are reported with and without the extended MHC region. The genomic inflation factor (1000) is 1.02.
(PDF)

S2 Fig Zoom plot for chromosome 4q12 locus harbouring *EXOC1*, previously reported to be associated with cervical cancer in Chinese. (PDF)

S3 Fig Zoom plot for chromosome 17q12 locus harbouring *GSDMB*, previously reported to be associated with cervical cancer in Chinese. (PDF)

S4 Fig Zoom plot for suggestive SNP rs3132461 association with cervical neoplasia. (PDF)

S5 Fig Zoom plot for suggestive SNP rs4396968 association with cervical neoplasia. (PDF)

S6 Fig Zoom plot for suggestive SNP rs7356297 association with cervical neoplasia. (PDF)

S7 Fig Zoom plot for suggestive SNP rs2267681 association with cervical neoplasia. (PDF)

S8 Fig Zoom plot for suggestive SNP rs56804039 association with cervical neoplasia. (PDF)

S9 Fig Zoom plot for suggestive SNP rs4738017 association with cervical neoplasia. (PDF)

S10 Fig Zoom plot for suggestive SNP rs9532669 association with cervical neoplasia. (PDF)

S11 Fig Zoom plot for suggestive SNP rs11637339 association with cervical neoplasia. (PDF)

S12 Fig Zoom plot for suggestive SNP rs17087933 association with cervical neoplasia. (PDF)

S13 Fig Manhattan plot of *P-*values for predictors in the optimised MultiBLUP model using 1341 cases and 3217 Controls with optimised parameters sig1 = 1e^−4^ and sig2 = 0.02. (PDF)
