# Correction: Defining the genetic susceptibility to cervical neoplasia—A genome-wide association study

**DOI:** 10.1371/journal.pgen.1007257

**Published:** 2018-03-01

**Authors:** Paul J. Leo, Margaret M. Madeleine, Sophia Wang, Stephen M. Schwartz, Felicity Newell, Ulrika Pettersson-Kymmer, Kari Hemminki, Goran Hallmans, Sven Tiews, Winfried Steinberg, Janet S. Rader, Felipe Castro, Mahboobeh Safaeian, Eduardo L. Franco, François Coutlée, Claes Ohlsson, Adrian Cortes, Mhairi Marshall, Pamela Mukhopadhyay, Katie Cremin, Lisa G. Johnson, Cornelia L. Trimble, Suzanne Garland, Sepehr N. Tabrizi, Nicolas Wentzensen, Freddy Sitas, Julian Little, Maggie Cruickshank, Ian H. Frazer, Allan Hildesheim, Matthew A. Brown

Dr. Cornelia L. Trimble is not included in the author byline. She should be listed as the twenty-second author and affiliated with Department of Gynecology and Obstetrics, Johns Hopkins University School of Medicine, Baltimore, Maryland, United States of America. The contributions of this author are as follows: Conceptualization, methodology and formal analysis.

The following information is missing from the Funding section: This study was supported by the Dana Foundation and the Maryland Cigarette Restitution Fund to CT and by the NCI grant 1K23CA85437.
