# Supplementary material for: Defining the genetic susceptibility to cervical neoplasia—A genome-wide association study
Source: PLoS Genet. 2017 Aug 14;13(8):e1006866. doi: 10.1371/journal.pgen.1006866 (PMC5570502; doi:10.1371/journal.pgen.1006866)
Supplement: S1 Table — The in phase alleles column denotes which alleles R2 terms refers where P = Present and A = absent; the term P/P denotes that the R2 is with respect to presence of the classical allele and the presence of the amino acids. (DOCX) [file pgen.1006866.s001.docx]

**Supplementary Table S1.** Amino-acid associations at amino-acid position 71 in HLA-DRB1. The in phase alleles column denotes which alleles R2 terms refers where P =Present and A=absent; the term P/P denotes that the R2 is with respect to presence of the classical allele and the presence of the amino acids.

| **Amino acid** | **FRQ** | **Odds-Ratio** | **SE** | ***P-*value** | **R2 with DRB1*1501** | **R2 with DRB1*1301** | **In Phase alleles DRB1*1501/AA** | **In Phase alleles DRB1*1301/AA** |
| --- | --- | --- | --- | --- | --- | --- | --- | --- |
| A | 0.15 | 1.42 | 0.05 | 1.44 × 10^−11^ | 0.963 | 0.011 | P/P | P/A |
| KA | 0.41 | 1.28 | 0.04 | 2.43 × 10^−10^ | 0.237 | 0.044 | P/P | P/A |
| K | 0.26 | 1.07 | 0.04 | 0.13 | 0.059 | 0.023 | P/A | P/A |
| KR | 0.73 | 0.99 | 0.04 | 0.75 | 0.412 | 0.161 | P/A | P/A |
| R | 0.47 | 0.94 | 0.04 | 0.097 | 0.135 | 0.053 | P/A | P/A |
| KE | 0.38 | 0.88 | 0.04 | 0.0013 | 0.111 | 0.095 | P/A | P/P |
| E | 0.12 | 0.67 | 0.06 | 2.57 × 10^−11^ | 0.027 | 0.395 | P/A | P/P |
