# Supplementary material for: Defining the genetic susceptibility to cervical neoplasia—A genome-wide association study
Source: PLoS Genet. 2017 Aug 14;13(8):e1006866. doi: 10.1371/journal.pgen.1006866 (PMC5570502; doi:10.1371/journal.pgen.1006866)
Supplement: S2 Table — The in phase alleles column denotes which alleles R2 terms refers where P = Present and A = absent; the term P/P denotes that the R2 is with respect to presence of the classical allele and the presence of the amino acids. (DOCX) [file pgen.1006866.s002.docx]

**Supplementary Table S2.** Amino-acid associations at amino-acid position 13 in HLA-DRB1. The in phase alleles column denotes which alleles R2 terms refers where P =Present and A=absent; the term P/P denotes that the R2 is with respect to presence of the classical allele and the presence of the amino acids.

| **Amino Acid** | **FRQ** | **Odds-Ratio** | **SE** | ***P-*value** | **R2 with DRB1*1501** | **R2 with DRB1*1301** | **In Phase alleles**  **AA/DRB1*1501** | **In Phase alleles**  **AA/DRB1*1301** |
| --- | --- | --- | --- | --- | --- | --- | --- | --- |
| R | 0.17 | 1.41 | 0.05 | 1.35 × 10^−11^ | 0.875 | 0.012 | P/P | P/A |
| RH | 0.37 | 1.37 | 0.04 | 1.11 × 10^−16^ | 0.295 | 0.036 | P/P | P/A |
| RG | 0.22 | 1.32 | 0.05 | 1.00 × 10^−9^ | 0.647 | 0.016 | P/P | P/A |
| RY | 0.29 | 1.22 | 0.04 | 1.17 × 10^−6^ | 0.409 | 0.026 | P/P | P/A |
| H | 0.21 | 1.17 | 0.04 | 1.80 × 10^−4^ | 0.041 | 0.016 | P/A | P/A |
| HG | 0.26 | 1.16 | 0.04 | 2.98 × 10^−4^ | 0.053 | 0.021 | P/A | P/A |
| RF | 0.31 | 1.14 | 0.04 | 0.0012 | 0.365 | 0.029 | P/P | P/A |
| SRH | 0.69 | 1.12 | 0.04 | 0.0076 | 0.079 | 0.031 | P/P | P/P |
| HY | 0.33 | 1.11 | 0.04 | 0.0058 | 0.080 | 0.031 | P/A | P/A |
| HF | 0.35 | 1.06 | 0.04 | 0.16 | 0.089 | 0.035 | P/A | P/A |
| G | 0.05 | 1.01 | 0.09 | 0.88 | 0.008 | 0.003 | P/A | P/A |
| YG | 0.18 | 0.97 | 0.05 | 0.55 | 0.034 | 0.013 | P/A | P/A |
| SRG | 0.53 | 0.97 | 0.04 | 0.42 | 0.152 | 0.059 | P/P | P/P |
| SR | 0.48 | 0.97 | 0.04 | 0.40 | 0.181 | 0.071 | P/P | P/P |
| Y | 0.13 | 0.96 | 0.06 | 0.47 | 0.024 | 0.009 | P/A | P/A |
| SRY | 0.61 | 0.95 | 0.04 | 0.16 | 0.108 | 0.042 | P/P | P/P |
| SH | 0.52 | 0.91 | 0.04 | 0.15 | 0.176 | 0.060 | P/A | P/P |
| SHG | 0.57 | 0.91 | 0.04 | 0.15 | 0.210 | 0.050 | P/A | P/P |
| SRF | 0.62 | 0.90 | 0.04 | 0.0061 | 0.097 | 0.038 | P/P | P/P |
| SHY | 0.64 | 0.89 | 0.04 | 0.0021 | 0.299 | 0.035 | P/A | P/P |
| YF | 0.27 | 0.88 | 0.04 | 0.0045 | 0.064 | 0.025 | P/A | P/A |
| FG | 0.19 | 0.88 | 0.05 | 0.012 | 0.040 | 0.016 | P/A | P/A |
| F | 0.14 | 0.84 | 0.06 | 0.0032 | 0.030 | 0.012 | P/A | P/A |
| SHF | 0.66 | 0.83 | 0.04 | 3.20 × 10^−6^ | 0.332 | 0.032 | P/A | P/P |
| SYG | 0.49 | 0.79 | 0.04 | 5.07 × 10^−10^ | 0.158 | 0.067 | P/A | P/P |
| SG | 0.36 | 0.79 | 0.04 | 2.87 × 10^−9^ | 0.093 | 0.113 | P/A | P/P |
| SY | 0.44 | 0.78 | 0.04 | 2.93 × 10^−10^ | 0.132 | 0.080 | P/A | P/P |
| S | 0.32 | 0.77 | 0.04 | 5.14 × 10^−10^ | 0.076 | 0.138 | P/A | P/P |
| SFG | 0.50 | 0.76 | 0.04 | 6.27 × 10^−14^ | 0.174 | 0.060 | P/A | P/P |
| SF | 0.46 | 0.75 | 0.04 | 1.99 × 10^−14^ | 0.146 | 0.072 | P/A | P/P |
| SYF | 0.58 | 0.73 | 0.04 | 1.92 × 10^−16^ | 0.245 | 0.043 | P/A | P/P |
