# Supplementary material for: Defining the genetic susceptibility to cervical neoplasia—A genome-wide association study
Source: PLoS Genet. 2017 Aug 14;13(8):e1006866. doi: 10.1371/journal.pgen.1006866 (PMC5570502; doi:10.1371/journal.pgen.1006866)
Supplement: S3 Table — Candidate genes are as reported in original reference. (DOCX) [file pgen.1006866.s003.docx]

**Supplementary Table S3.** GWAS findings at loci previously reported to be associated with cervical cancer. Candidate genes are as reported in original reference.

| **SNP** | **Chromosome** | **Position** | **Candidate gene at locus** | **Risk Allele** | **OR** | ***P-*value** | **Reference** |
| --- | --- | --- | --- | --- | --- | --- | --- |
| rs4297265 | 1 | 67852335 | *IL12RB2* | G | 0.92 | 0.02 | [1] |
| rs2229546 | 1 | 67861520 | *IL12RB2* | C | 0.94 | 0.09 | [1] |
| rs4149963 | 1 | 242035382 | *EXO1* | T | 1.10 | 0.12 | [2] |
| rs13117307 | 4 | 56751740 | *EXOC1* | T | 1.01 | 0.79 | [3] |
| rs3804100 | 4 | 154625409 | *TLR2* | C | 1.01 | 0.93 | [2] |
| rs401681 | 5 | 1322087 | *TERT* | T | 0.98 | 0.48 | [4] |
| rs26653 | 5 | 96139250 | *ERAP1* | C | 0.97 | 0.46 | [5] |
| rs27024 | 5 | 97959361 | *ERAP1* | A | 0.98 | 0.63 | [5] |
| rs3181224 | 5 | 158740850 | *IL12B* | G | 0.94 | 0.30 | [1] |
| rs853360 | 6 | 14134592 | *CD83* | T | 1.00 | 0.91 | [6] |
| rs2894054 | 6 | 30872274 | *GTF2H4* | NA^#^ | | | [7] |
| rs114596632  (rs1264308) | 6 | 30879987 | *GTF2H4* | T | 0.94 | 0.24^*^ | [7] |
| rs67841474 | 6 | 31272182 | *MICA* | G | 1.00 | 0.97 | [8] |
| rs2239704 | 6 | 31540141 | *TNF* | A | 1.23 | 5.65 × 10^−9^ | [6] |
| rs2229094 | 6 | 31540556 | *TNF* | C | 0.85 | 0.0001 | [6] |
| rs1799964 | 6 | 31542308 | *TNF* | C | 0.86 | 0.0006 | [6] |
| rs9272143 | 6 | 32600803 | *HLA-DRB1* | C | 0.74 | 5.2 × 10^−15^ | [8] |
| rs4148876 | 6 | 32796793 | *TAP2* | A | 1.00 | 0.97 | [5] |
| rs2071543 | 6 | 32811629 | *LMP7* | T | 1.05 | 0.37 | [5] |
| rs4737999 | 8 | 70518035 | *SULF1* | A | 0.97 | 0.50 | [7] |
| rs4252314 | 11 | 117868517 | *IL10RA* | G | 1.09 | 0.36 | [1] |
| rs9610 | 11 | 117872086 | *IL10RA* | A | 0.98 | 0.59 | [1] |
| rs11177074 | 12 | 68544434 | *IFNG* | C | 0.93 | 0.33 | [7] |
| rs12302655 | 12 | 113374506 | *OAS3* | NA^#^ | | | [7] |
| rs7138267 | 12 | 113409981 |  | T | 0.92 | 0.03^*^ |  |
| rs3784621 | 15 | 48633092 | *DUT* | C | 0.95 | 0.33 | [7] |
| rs7195830 | 16 | 88709712 | *CYBA* | A | 1.02 | 0.58 | [2] |
| rs2239359 | 16 | 89849480 | *FANCA* | T | 1.02 | 0.65 | [2] |
| rs1042522 | 17 | 7579472 | *TP53* | G | 1.01 | 0.75 | [9] |
| rs8067378 | 17 | 38051348 | *GSDMB* | A | 1.02 | 0.67 | [3] |
| rs412611 | 17 | 76132259 | *EVER1/2* | T | 1.2 | 0.01* | [7] |
| rs9893818 | 17 | 76142167 | *EVER1/2* | NA^#^ | | | [7] |
| rs11575934 | 19 | 18186618 | *IL12RB1* | NA# | | | [1] |
| rs8105810 | 19 | 18189255 | *IL12RB1* | C | 0.88 | 0.006* | [1] |
| rs25487 | 19 | 44055726 | *XRCC1* | T | 0.97 | 0.37 | [2] |
| rs7251 | 19 | 50162909 | *IRF3* | G | 0.92 | 0.05 | [2] |
| rs5757133 | 22 | 38947835 | *DMC1* | T | 0.94 | 0.13 | [7] |

^#^Did not pass quality filtering or was not imputed

^*^Lowest *P-*value in candidate gene

**Supplementary Table S3 References**

1. Hussain SK, Madeleine MM, Johnson LG, Du Q, Galloway DA, Daling JR, et al. Nucleotide variation in IL-10 and IL-12 and their receptors and cervical and vulvar cancer risk: a hybrid case-parent triad and case-control study. International journal of cancer Journal international du cancer. 2013;133(1):201-213.

2. Wang SS, Bratti MC, Rodriguez AC, Herrero R, Burk RD, Porras C, et al. Common variants in immune and DNA repair genes and risk for human papillomavirus persistence and progression to cervical cancer. The Journal of infectious diseases. 2009;199(1):20-30.

3. Shi Y, Li L, Hu Z, Li S, Wang S, Liu J, et al. A genome-wide association study identifies two new cervical cancer susceptibility loci at 4q12 and 17q12. Nature genetics. 2013;45(8):918-922.

4. Rafnar T, Sulem P, Stacey SN, Geller F, Gudmundsson J, Sigurdsson A, et al. Sequence variants at the TERT-CLPTM1L locus associate with many cancer types. Nature genetics. 2009;41(2):221-227.

5. Mehta AM, Jordanova ES, van Wezel T, Uh HW, Corver WE, Kwappenberg KM, et al. Genetic variation of antigen processing machinery components and association with cervical carcinoma. Genes, chromosomes & cancer. 2007;46(6):577-586.

6. Bodelon C, Madeleine MM, Johnson LG, Du Q, Galloway DA, Malkki M, et al. Genetic variation in the TLR and NF-kappaB pathways and cervical and vulvar cancer risk: a population-based case-control study. International journal of cancer Journal international du cancer. 2014;134(2):437-444.

7. Wang SS, Gonzalez P, Yu K, Porras C, Li Q, Safaeian M, et al. Common genetic variants and risk for HPV persistence and progression to cervical cancer. PloS one. 2010;5(1):e8667.

8. Chen D, Hammer J, Lindquist D, Idahl A, Gyllensten U. A variant upstream of HLA-DRB1 and multiple variants in MICA influence susceptibility to cervical cancer in a Swedish population. Cancer medicine. 2014;3(1):190-198.

9. Klug SJ, Ressing M, Koenig J, Abba MC, Agorastos T, Brenna SM, et al. TP53 codon 72 polymorphism and cervical cancer: a pooled analysis of individual data from 49 studies. The Lancet Oncology. 2009;10(8):772-784.
