# Supplementary material for: Defining the genetic susceptibility to cervical neoplasia—A genome-wide association study
Source: PLoS Genet. 2017 Aug 14;13(8):e1006866. doi: 10.1371/journal.pgen.1006866 (PMC5570502; doi:10.1371/journal.pgen.1006866)
Supplement: S4 Table — (DOCX) [file pgen.1006866.s004.docx]

**Supplementary Table S4.** Non-MHC SNPs achieving suggestive (5 × 10^−8^ < *P* <10^−5^) association with cervical neoplasia.

| **SNP** | **Chromosome** | **Position** | **Candidate gene at locus** | **Risk Allele** | **OR** | ***P-*value** |
| --- | --- | --- | --- | --- | --- | --- |
| rs4396968 | 4 | 47089184 | *GABRB1* | T | 1.26 | 1.21 × 10^−6^ |
| rs7356297 | 4 | 90746329 | *SNCA* | C | 0.8603 | 2.27 × 10^−5^ |
| rs3132461 | 6 | 31480668 | Intergenic *(MICB,MCCD1)* | G | 1.358 | 1.55 × 10^−13^ |
| rs2267681 | 7 | 138994119 | *TBXAS1* | G | 0.847 | 1.91 × 10^−6^ |
| rs56804039 | 8 | 8381029 | Intergenic *(SGK223,CLDN23)* | G | 0.827 | 4.40 × 10^−6^ |
| rs4738017 | 8 | 70576130 | Intergenic *(SULF1,SLCO5A1)* | G | 1.174 | 7.34 × 10^−6^ |
| rs9532669 | 13 | 41501550 | Intergenic *(SUGT1P3,ELF1)* | A | 1.216 | 1.71 × 10^−7^ |
| rs11637339 | 15 | 99992008 | Intergenic *(LRRC28,MEF2A)* | C | 1.184 | 5.50 × 10^−6^ |
| rs17087933 | 18 | 71263350 | Intergenic *(LOC100505817,FBXO15)* | G | 0.782 | 1.66 × 10^−6^ |
