# Supplementary material for: Defining the genetic susceptibility to cervical neoplasia—A genome-wide association study
Source: PLoS Genet. 2017 Aug 14;13(8):e1006866. doi: 10.1371/journal.pgen.1006866 (PMC5570502; doi:10.1371/journal.pgen.1006866)
Supplement: S1 Fig — Findings are reported with and without the extended MHC region. The genomic inflation factor (1000) is 1.02. (PDF) [file pgen.1006866.s005.pdf]

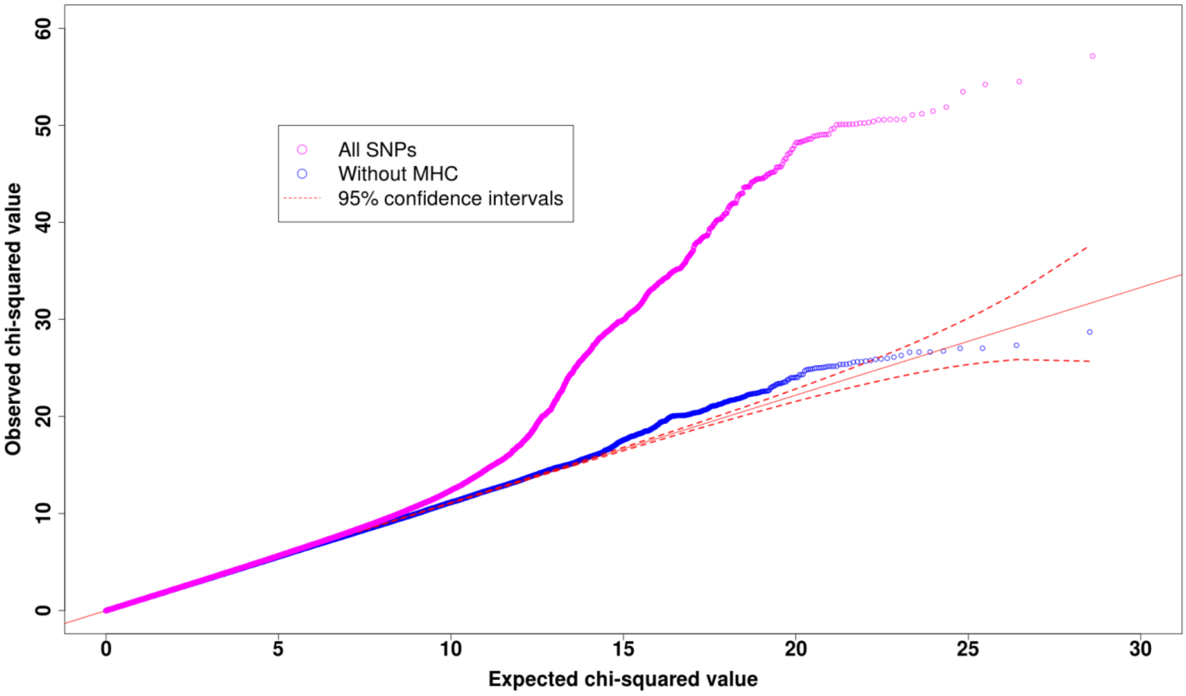

Supplementary Figure S1. Q-Q plot for overall association findings. Findings are reported with and without the extended MHC region. The genomic inflation factor (1000) is 1.02.
