# Supplementary material for: Defining the genetic susceptibility to cervical neoplasia—A genome-wide association study
Source: PLoS Genet. 2017 Aug 14;13(8):e1006866. doi: 10.1371/journal.pgen.1006866 (PMC5570502; doi:10.1371/journal.pgen.1006866)
Supplement: S2 Fig — (PDF) [file pgen.1006866.s006.pdf]

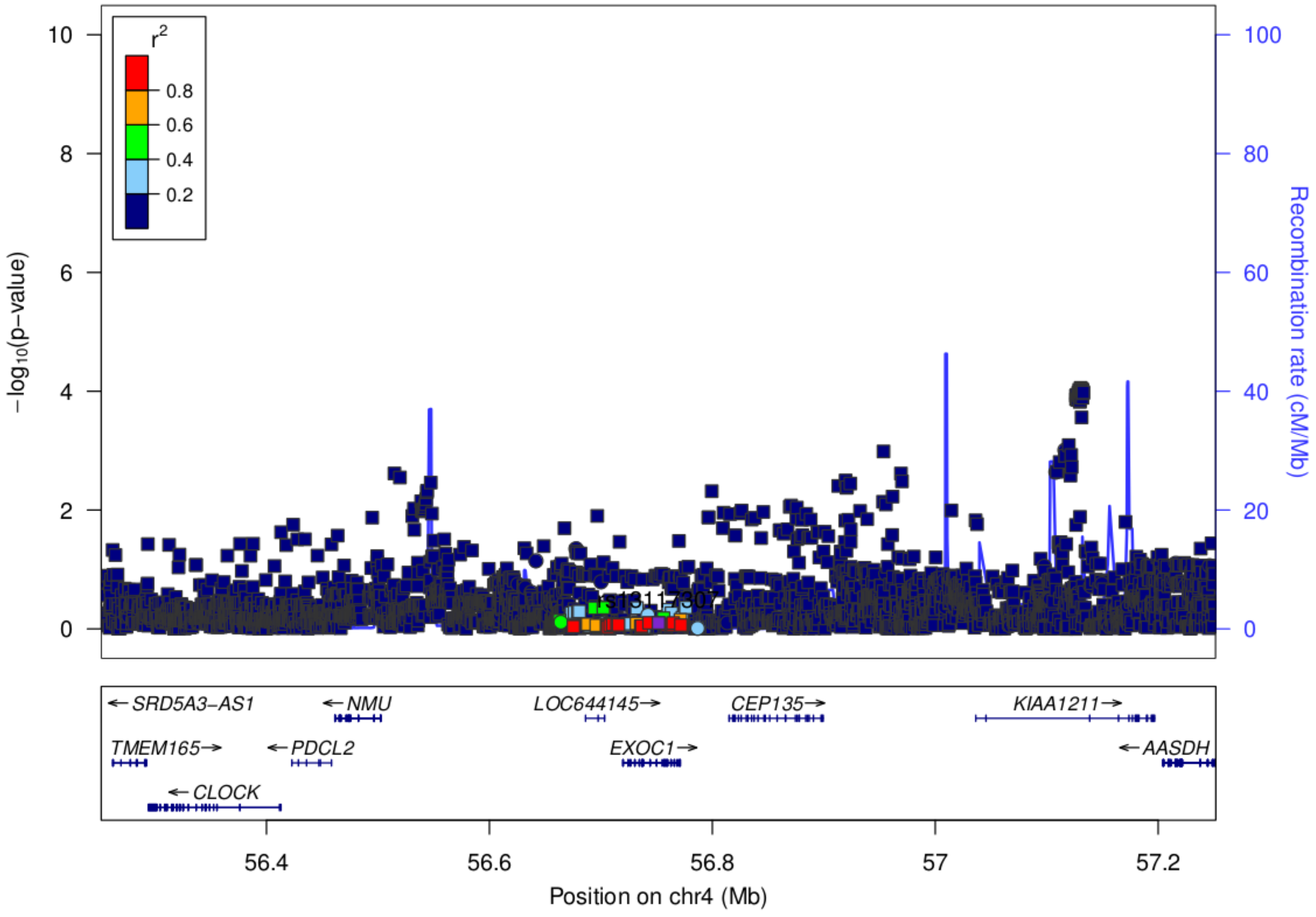

Supplementary Figure S2. Zoom plot for chromosome 4q12 locus harbouring *EXOC1*, previously reported to be associated with cervical cancer in Chinese.
