# Supplementary figures and images for: Defining the genetic susceptibility to cervical neoplasia—A genome-wide association study
Source: PLoS Genet. 2017 Aug 14;13(8):e1006866. doi: 10.1371/journal.pgen.1006866 (PMC5570502; doi:10.1371/journal.pgen.1006866)

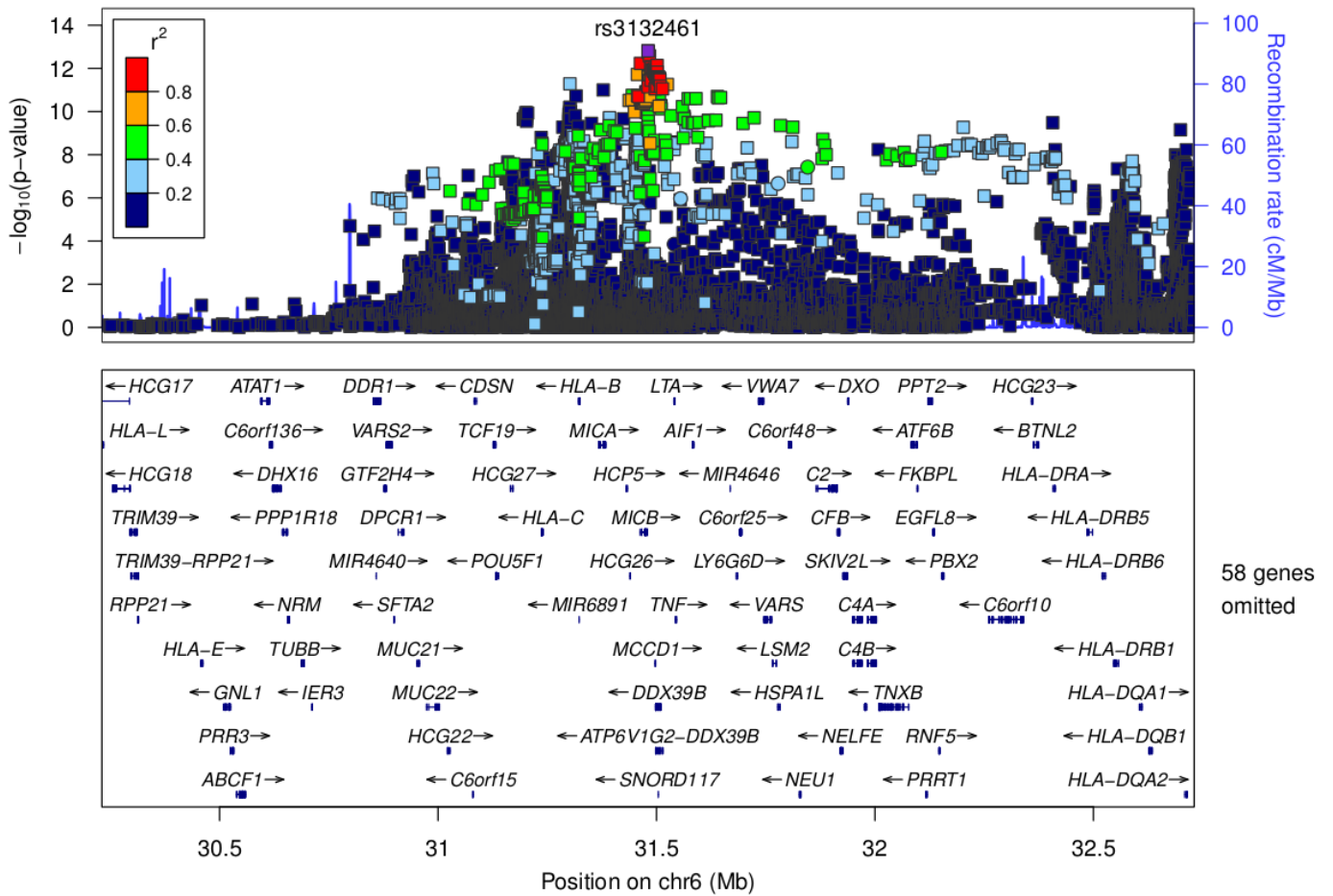

**Supplementary Figure S4.** Zoom plot for suggestive SNP rs3132461 association with cervical neoplasia.

Supplement: S4 Fig — (PDF) [file pgen.1006866.s008.pdf]

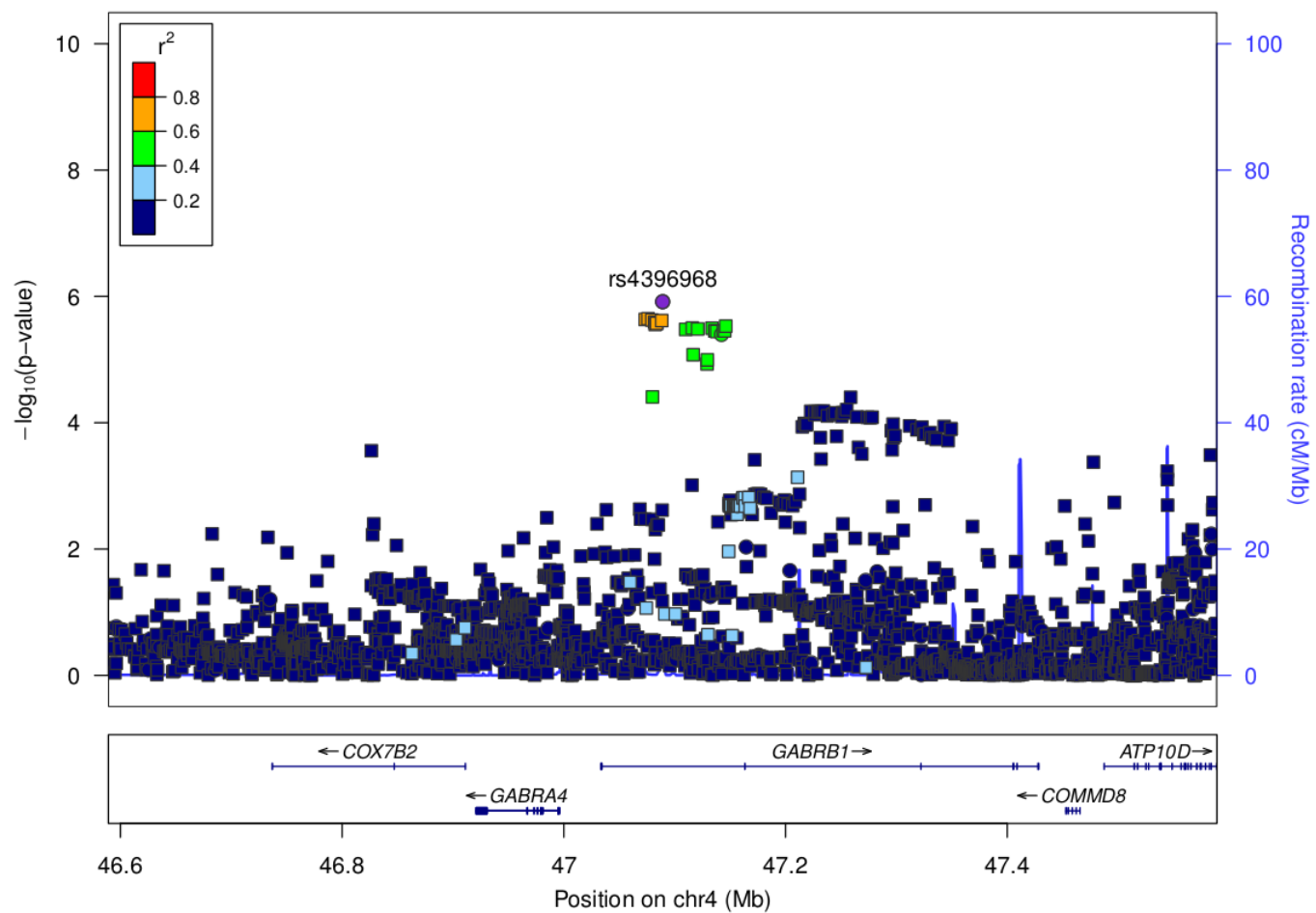

**Supplementary Figure S5.** Zoom plot for suggestive SNP rs4396968 association with cervical neoplasia.

Supplement: S5 Fig — (PDF) [file pgen.1006866.s009.pdf]

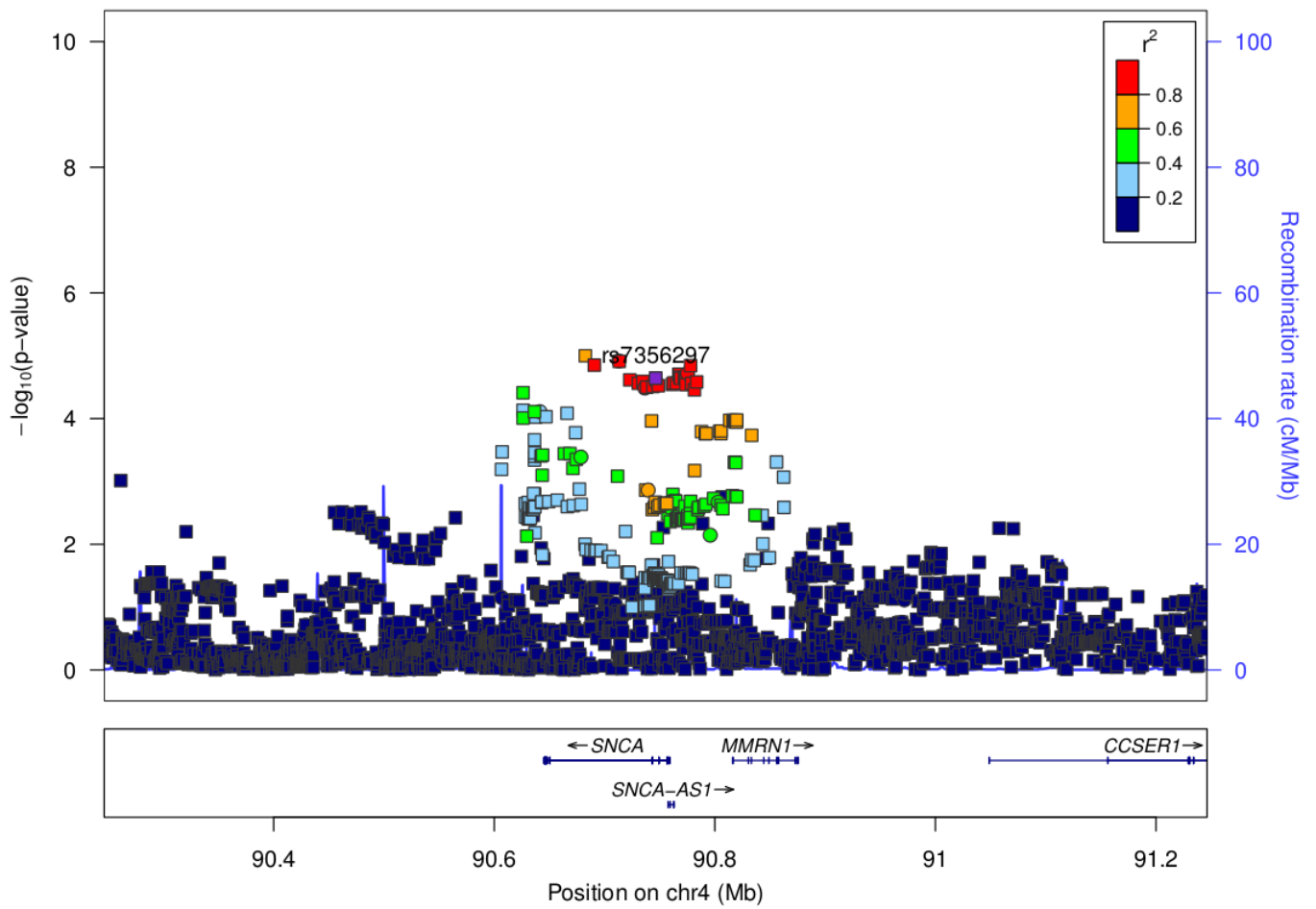

**Supplementary Figure S6.** Zoom plot for suggestive SNP rs7356297 association with cervical neoplasia.

Supplement: S6 Fig — (PDF) [file pgen.1006866.s010.pdf]

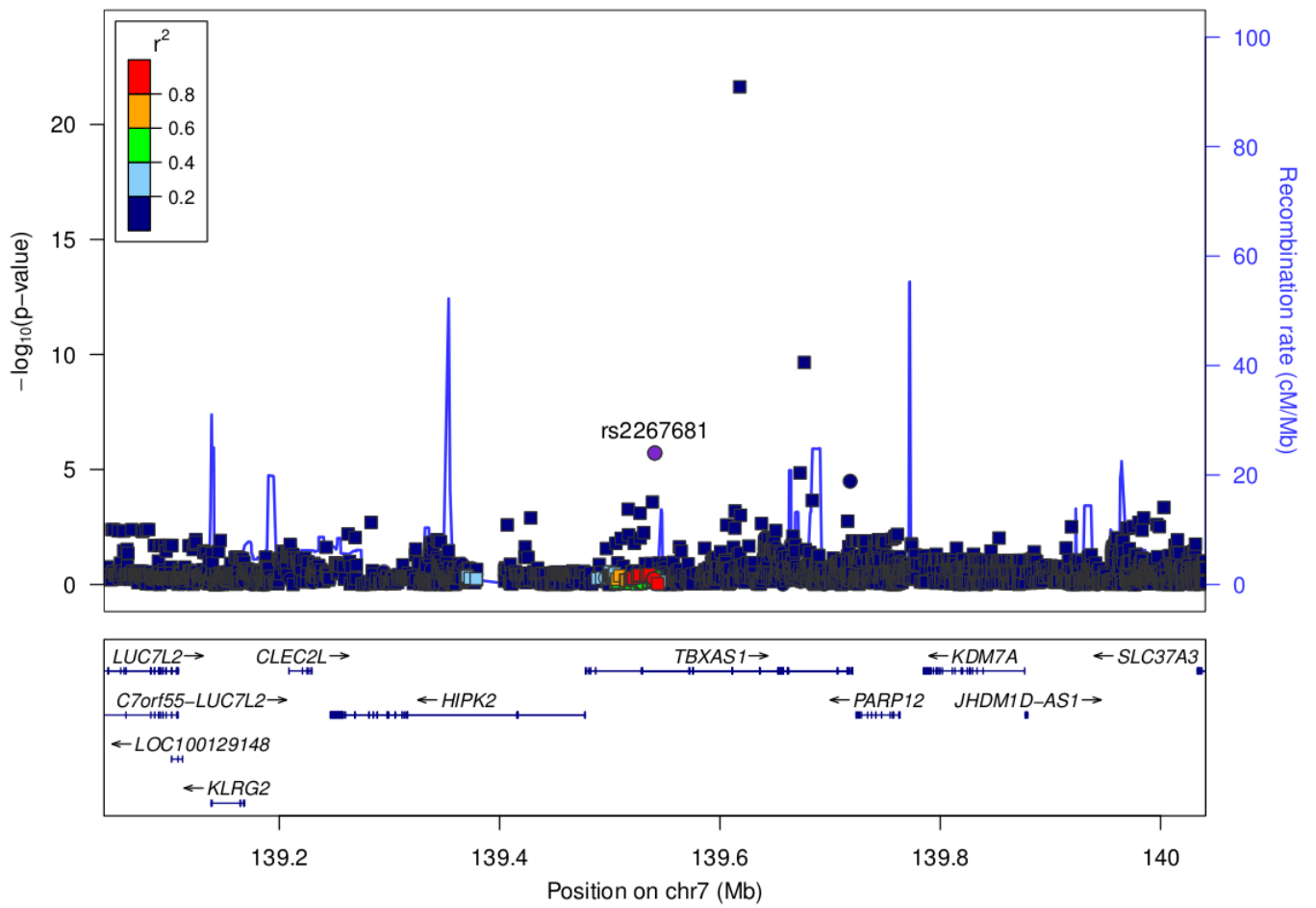

**Supplementary Figure S7.** Zoom plot for suggestive SNP rs2267681 association with cervical neoplasia.

Supplement: S7 Fig — (PDF) [file pgen.1006866.s011.pdf]

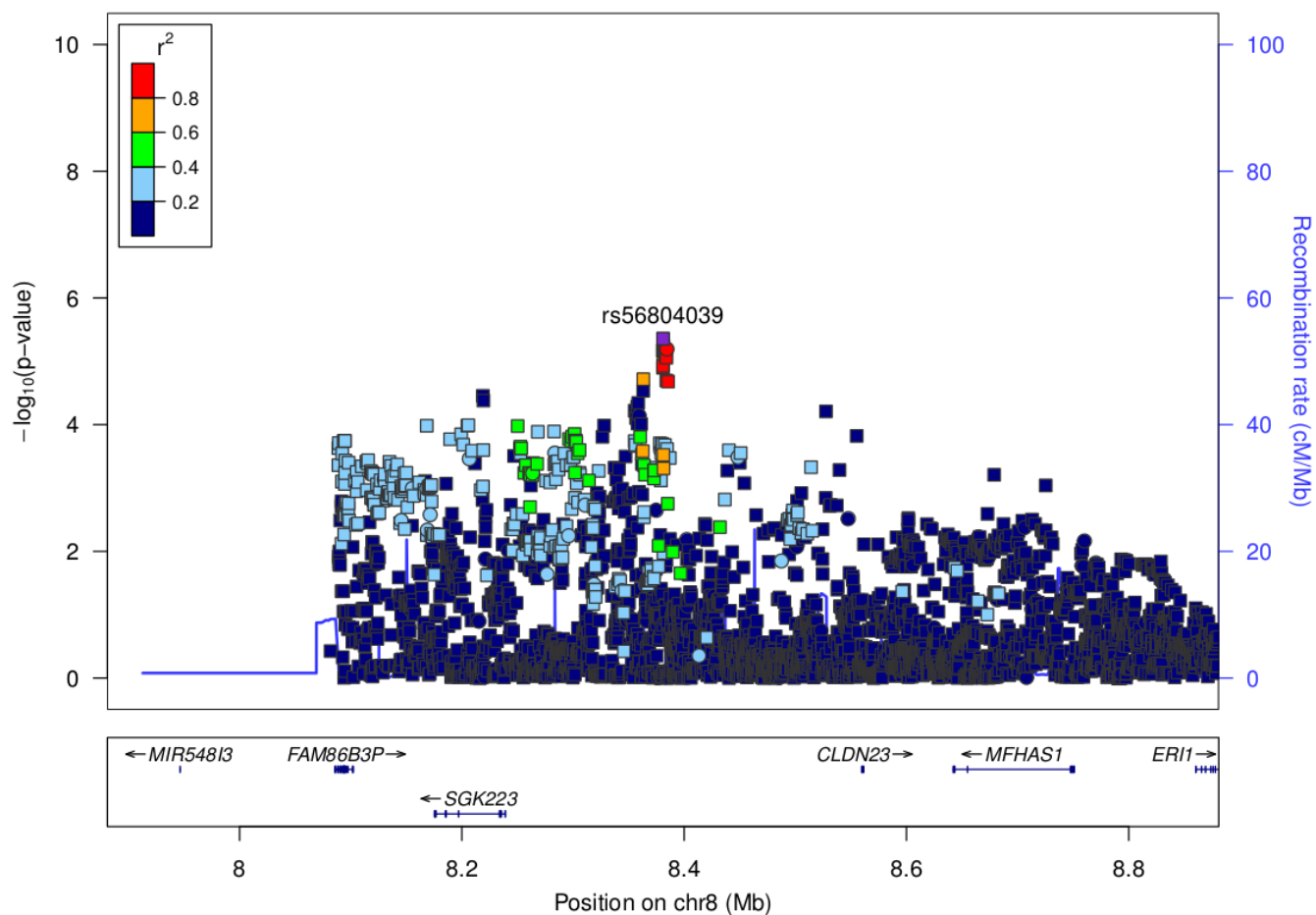

**Supplementary Figure S8.** Zoom plot for suggestive SNP rs56804039 association with cervical neoplasia.

Supplement: S8 Fig — (PDF) [file pgen.1006866.s012.pdf]

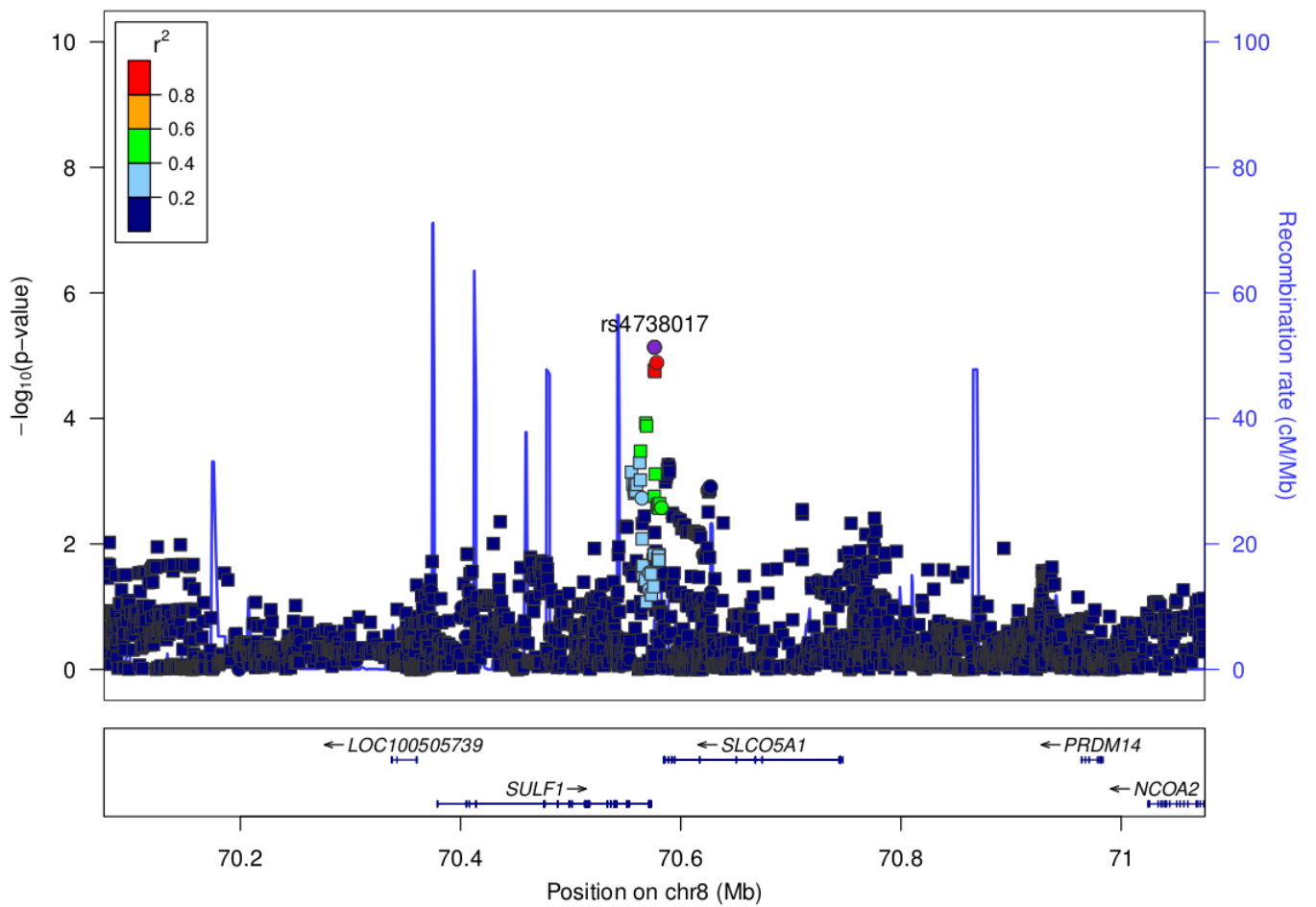

**Supplementary Figure S9.** Zoom plot for suggestive SNP rs4738017 association with cervical neoplasia.

Supplement: S9 Fig — (PDF) [file pgen.1006866.s013.pdf]

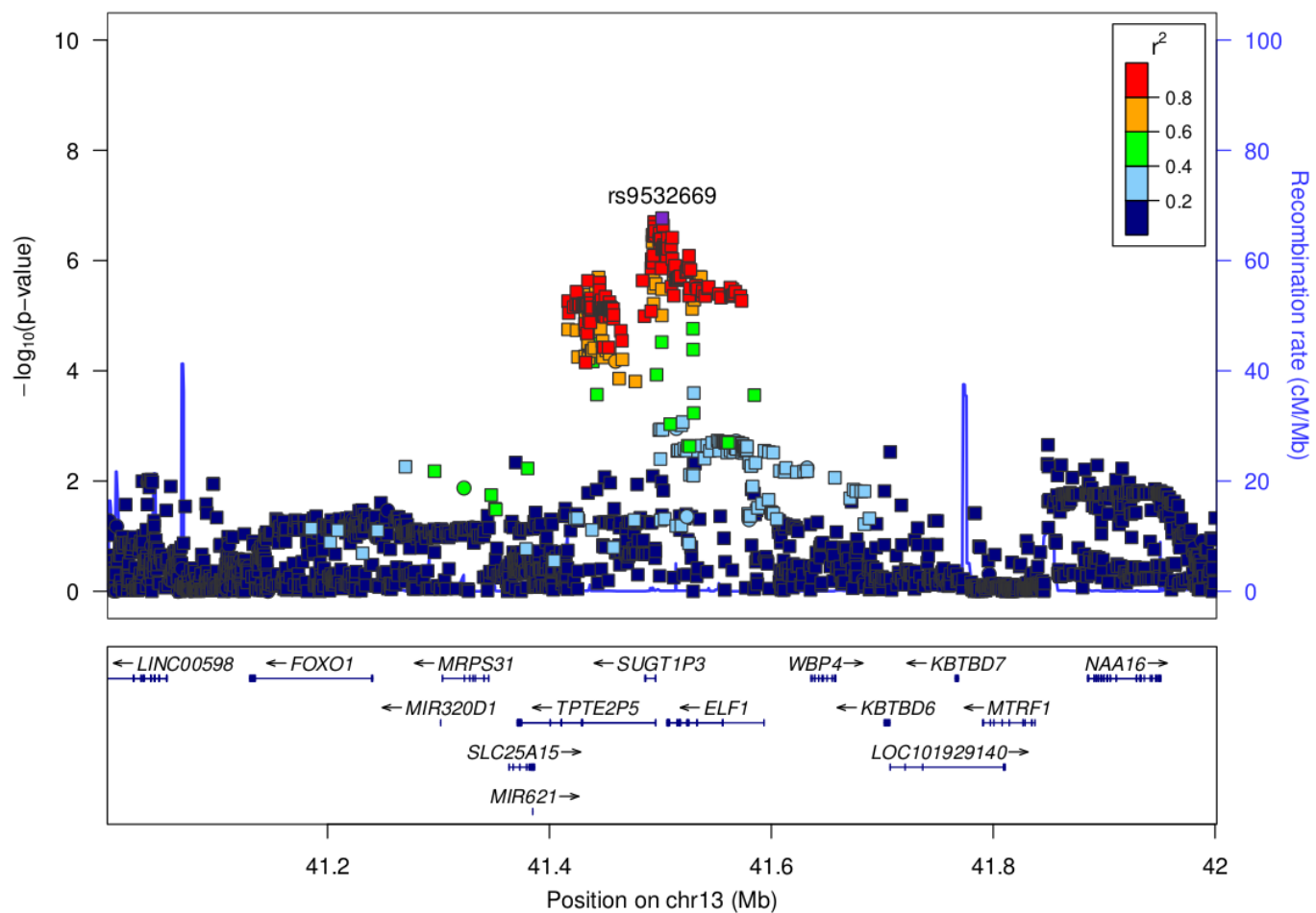

**Supplementary Figure S10.** Zoom plot for suggestive SNP rs9532669 association with cervical neoplasia.

Supplement: S10 Fig — (PDF) [file pgen.1006866.s014.pdf]

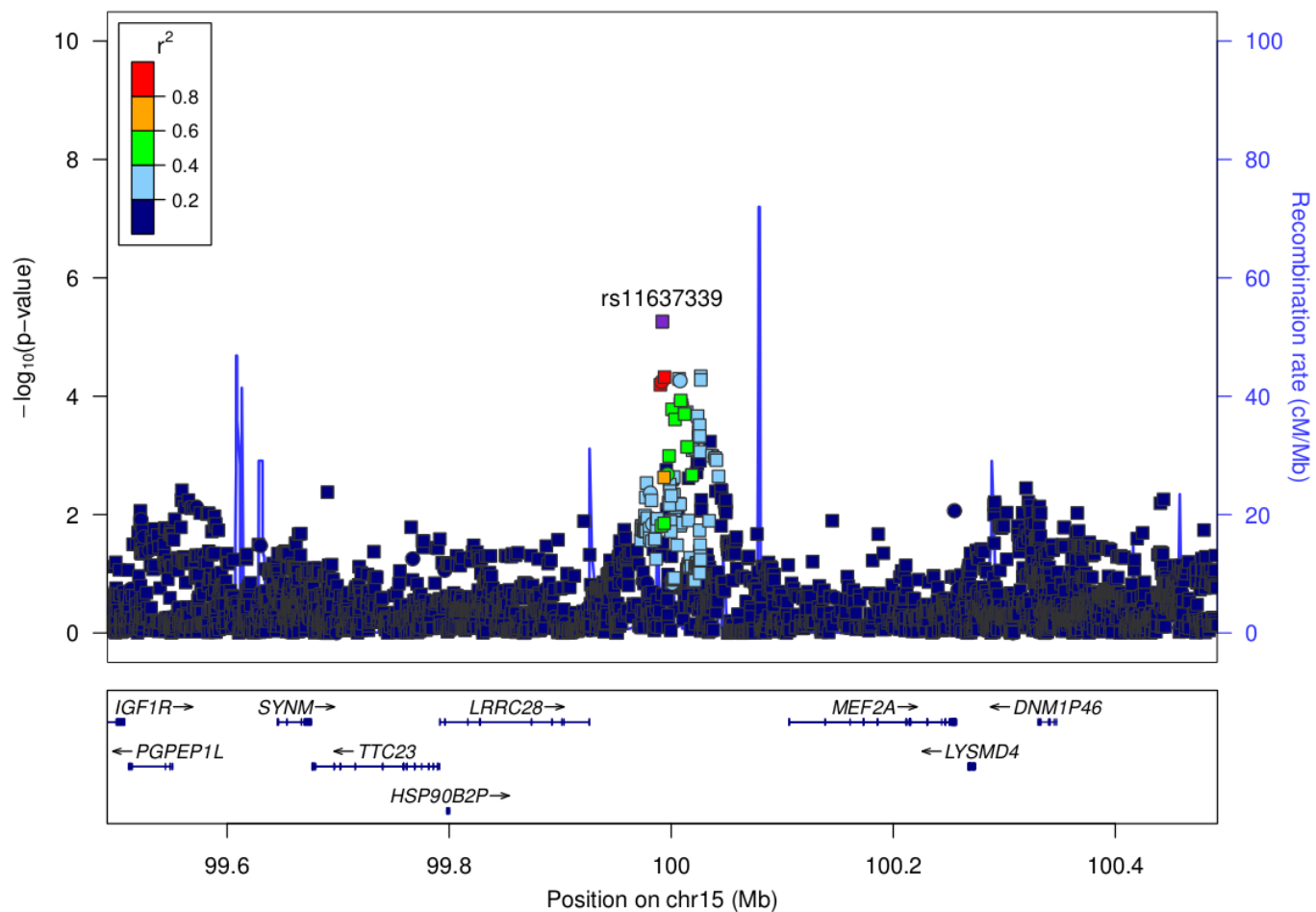

**Supplementary Figure S11.** Zoom plot for suggestive SNP rs11637339 association with cervical neoplasia.

Supplement: S11 Fig — (PDF) [file pgen.1006866.s015.pdf]

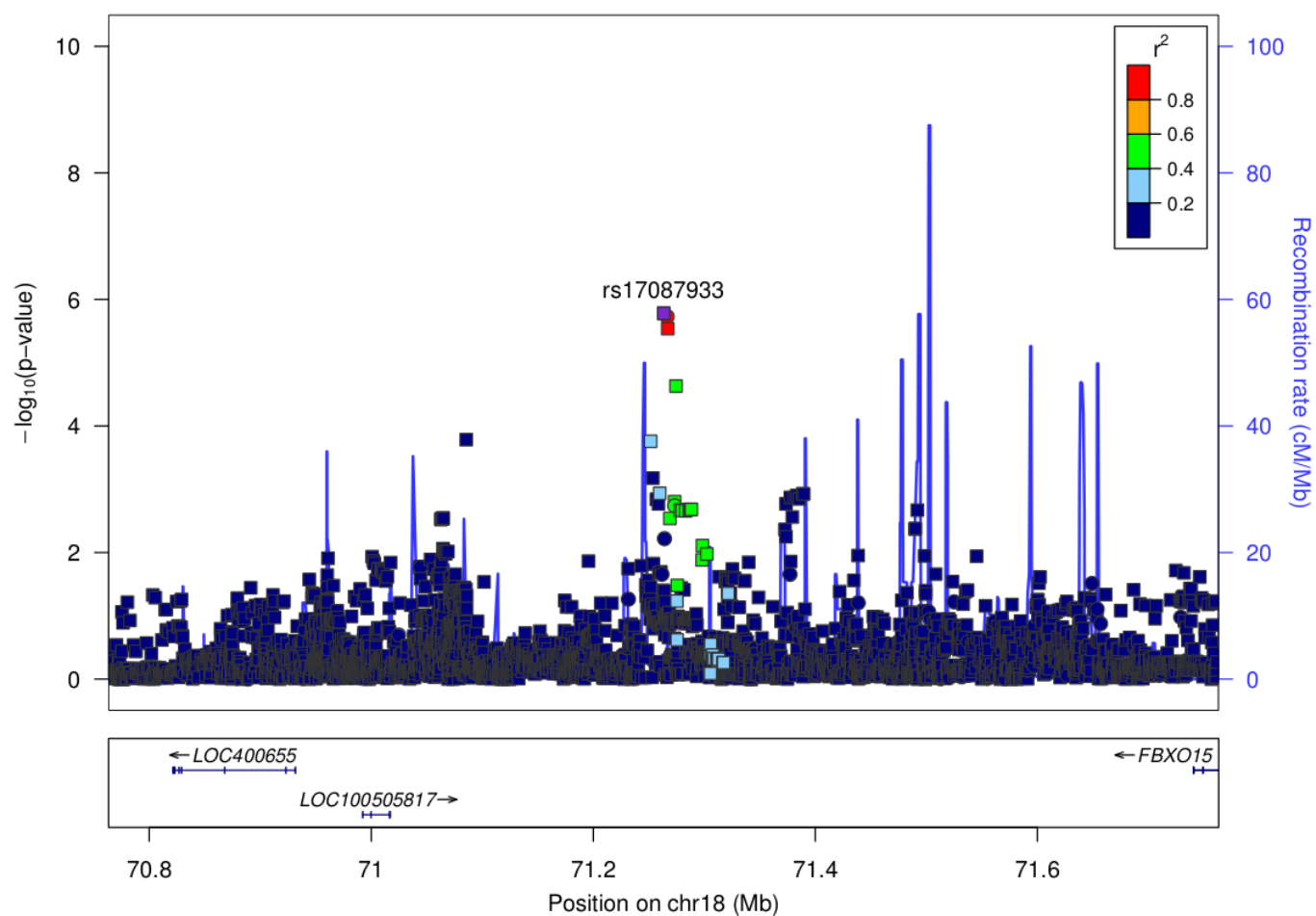

**Supplementary Figure S12.** Zoom plot for suggestive SNP rs17087933 association with cervical neoplasia.

Supplement: S12 Fig — (PDF) [file pgen.1006866.s016.pdf]
