# Supplementary material for: Defining the genetic susceptibility to cervical neoplasia—A genome-wide association study
Source: PLoS Genet. 2017 Aug 14;13(8):e1006866. doi: 10.1371/journal.pgen.1006866 (PMC5570502; doi:10.1371/journal.pgen.1006866)
Supplement: S13 Fig — (PDF) [file pgen.1006866.s017.pdf]

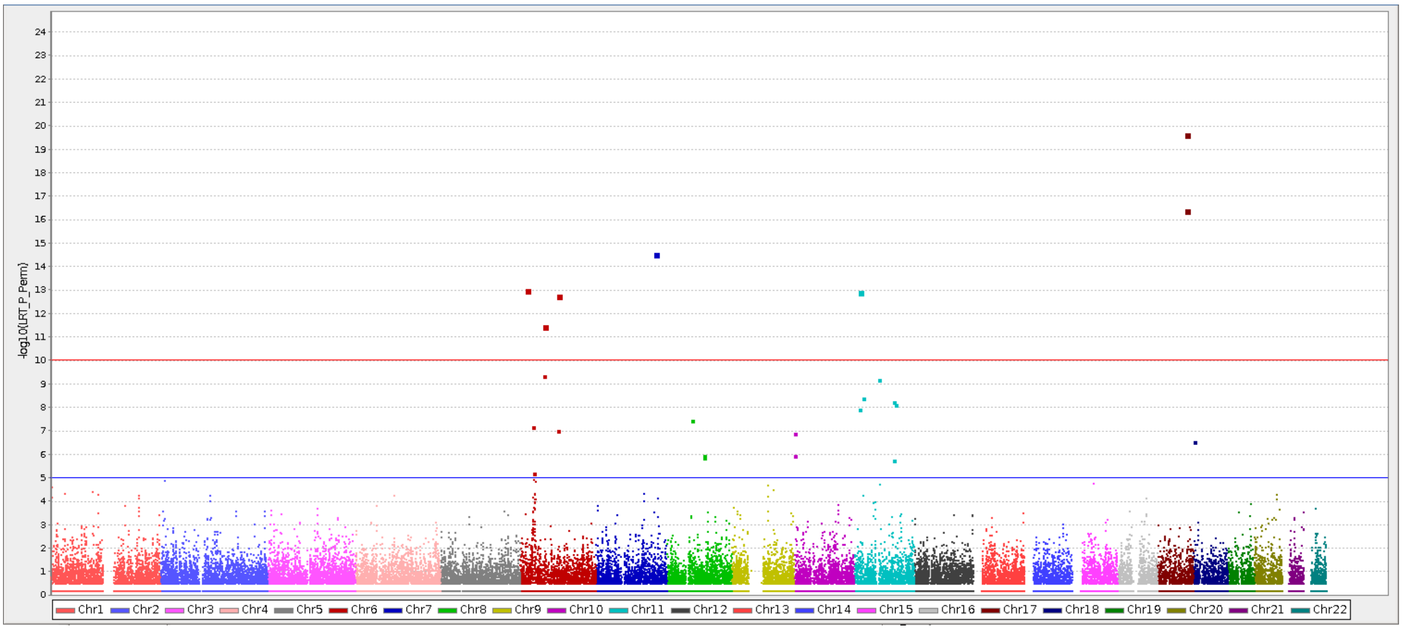

Supplementary Figure S13. Manhattan plot of *P*-values for predictors in the optimised MultiBLUP model using 1341 cases and 3217 Controls with optimised parameters sig1=1e^{-4} and sig2=0.02.
